# Electrosynthesis and Electrochromism of a New Crosslinked Polydithienylpyrrole with Diphenylpyrenylamine Subunits

**DOI:** 10.3390/polym12122777

**Published:** 2020-11-24

**Authors:** Yu-Ruei Kung, Sin-Yun Cao, Sheng-Huei Hsiao

**Affiliations:** 1 Department of Chemical Engineering and Biotechnology, Tatung University, Taipei 10452, Taiwan; 2 Department of Chemical Engineering and Biotechnology, National Taipei University of Technology, Taipei 10608, Taiwan; a0981363352@gmail.com

**Keywords:** conjugated polymers, electrochromic polymers, 2,5-Di(2-thienyl)-1*H*-pyrrole, diphenylpyrenylamine

## Abstract

A new electroactive monomer with two 2,5-di(2-thienyl)pyrrole (SNS) units and one diphenylpyrenylamine (DPPA) subunit, namely *N*,*N*-bis(4-(2,5-di(2-thienyl)-1*H*-pyrrol-1-yl)-phenyl)-1-aminopyrene (DPPA-2SNS), was synthesized from 1,4-di-(2-thienyl)butane-1,4-dione with *N*,*N*-di(4-aminophenyl)-1-aminopyrene through the Paal–Knorr condensation reaction. Visible and near-infrared (NIR) electrochromic polymer films could be facilely generated on the ITO-glass surface by the electrochemical polymerization of DPPA-2SNS in an electrolyte solution. The electro-synthesized polymer films exhibit multi-staged redox processes and multi-colored anodic electrochromic behavior. A multi-colored electrochromism, with yellowish orange, greyish blue, and purplish black colors, was observed in the polymer film by applying a positive potential. The polymer films exhibit reasonable coloration efficiency, fast response time, and good cycling stability, especially when switched between neutral and the first oxidation states. For comparison, *N*-(1-pyrenyl)-2,5-di(2-thienyl)pyrrole (Py-SNS) was also prepared and characterized with electrochemical and electro-optical properties.

## 1. Introduction

Electrochromism (EC) refers to a reversible change in the optical absorption or color of electroactive species as a result of electrochemical oxidation or reduction induced by a simple electric potential [1]. Over the past two decades, EC materials have received great interest and have been extensively investigated for many potential applications [2,3,4,5,6,7], such as smart windows, displays, automatically dimming mirrors, e-paper, self-tunable eyewear, adaptive camouflage, and energy storage devices. There are many types of EC materials that have been developed, including inorganic metal oxides, organic small molecules, organic π-conjugated polymers, and organic–inorganic coordination polymers [8]. In general, organic π-conjugated polymers demonstrate a number of advantages over inorganic and organic small molecule materials [9,10,11,12]. They possess great potential for the fabrication of flexible devices, diverse and multicolored electrochromisms, facile molecular design, and high coloration efficiency.

Triphenylamine (TPA) and its derivatives are the important building blocks for the preparation of electroactive molecules because of their excellent electron donating nature, the easy oxidizability of their nitrogen center, and their ability to transport charge carriers with high stability [13,14,15]. Many triarylamine derivatives and polymers have been developed for potential optoelectronic applications such as organic light-emitting devices (OLEDs), organic field-effect transistors (OFETs), and solar cells [16,17,18]. Triarylamine-containing condensation polymers such as aromatic polyamides and polyimides have been developed as a type of promising high-performance functional material owing to several useful properties such as excellent thermal stability, high electrochemical stability, good electrochromic performance, and the formation of stable amorphous films for application in thin-film devices [19,20].

The electronic and photophysical properties of pyrene are very attractive for the design of new organic light-emitting and semiconductor materials. The long fluorescence lifetime, large Stokes shift, and the tunable intensity of the excimer make the pyrene unit a useful form of fluorophore labeling in metal ion or nucleic acid probes [21,22]. The derivatives, polymers, starbursts, and dendrimers of pyrene have been extensively investigated for opto-electronic applications such as OLEDs, because of their emissive properties combined with high charge carrier mobility [23]. Owing to the attractive properties associated with the pyrene and triarylamine moieties, we have developed many diphenylpyrenylamine (DPPA)-based polymers with dual fluorescent and electrochromic functions [24,25,26].

Thiophene, pyrrole, carbazole, viologen, triphenylamine and others are commonly used as building blocks in order to modify the electrochromic properties of the π-conjugated polymers [27,28,29,30,31,32]. Since 1986, poly(2,5-dithienylpyrrole)s (PSNS) have attracted particular interest, which could be easily synthesized via chemical and electrochemical polymerization of 2,5-dithienylpyrrole (SNS) unit [33,34,35]. In the past three decades, an exciting class of conducting polymer, PSNS derivatives, have been much investigated for their electrochromic properties [36,37,38,39,40,41,42]. Because of their thiophene–pyrrole–thiophene terarylenic structures, all the monomers and their corresponding polymers exhibited low oxidation potential. In general, the PSNS homopolymers displayed a yellow to blue coloration with a short switching time. In addition, the electrochemical and electrochromic properties can be easily adjusted by attaching a second electroactive moiety such as triarylamine on the side chain [43,44,45].

In this work, a new electropolymerizable monomer containing a diphenylpyrenylamine (DPPA) core and two SNS units, coded as DPPA-2SNS, was prepared from *N*,*N*-di(4-aminophenyl)-1-aminopyrene with two equivalent amounts of 1,4-di(2-thienyl)butane-1,4-dione via the Paal–Knorr pyrrole synthesis. The electrochemical and optical properties of this SNS monomer were investigated. Their corresponding PSNS films were prepared by electropolymerization, and their electrochemical, electrochromic, and fluorescent properties were investigated and compared to those from *N*-(1-pyrenyl)-2,5-di(2-thienyl)pyrrole (Py-SNS), which has been reported in the literature [46].

## 2. Experimental Section

### 2.1. Materials

The synthetic details and characterization data of the starting materials including 1-aminopyrene, *N*,*N*-di(4-aminophenyl)-1-aminopyrene (DPPA-2NH_2_) have been reported previously [24]. The synthetic details and analysis of 1,4-di(2-thienyl)butane-1,4-dione (DTBDO), *N*-(1-pyrenyl)-2,5-di(2-thienyl)pyrrole (Py-SNS), *N*,*N*-bis(4-(2,5-di(2-thienyl)-1*H*-pyrrol-1-yl)phenyl)-1-aminopyrene (DPPA-2SNS) were summarized in the Supplementary Materials.

### 2.2. Electrochemical Polymerization

Electrochemical polymerization was performed with a CH Instruments 750A electrochemical analyzer. The PSNS were prepared by repetitive CV cycling of the SNS monomers in acetonitrile (MeCN) or dichloromethane (CH_2_Cl_2_) solutions containing 0.1 M Bu_4_NClO_4_ at a scan rate of 50 mV/s in a suitable potential range for ten cycles. The polymer was deposited onto the surface of the working electrode (ITO/glass substrate, polymer films area about 0.8 × 2.0 cm^2^), and the film was rinsed with plenty of acetone and dichloromethane for the removal of the un-reacted monomer, inorganic salts and other organic impurities formed during the process.

### 2.3. Measurements and Methods

Infrared (IR) spectra were recorded on a Horiba FT-720 FT-IR spectrometer. ^1^H NMR spectra were measured on a Bruker Avance III HD-600 MHz NMR spectrometer with tetramethylsilane (TMS) as an internal standard. Ultraviolet-visible (UV-Vis) spectra of the synthesized compounds and polymer films were recorded on an Agilent 8454 UV-visible spectrometer. Electrochemistry was performed with a CHI 750A electrochemical analyzer (Austin, TX, USA). Voltammograms are presented with the positive potential pointing to the left and with increasing anodic currents pointing downwards. Cyclic voltammetry (CV) was conducted with the use of a three-electrode cell in which ITO (polymer films area about 0.8 × 2.0 cm^2^) was used as a working electrode. Ferrocene was used as an external reference for calibration (+0.48 V vs. Ag/AgCl). Spectroelectrochemistry analyses were carried out with an electrolytic cell, which was composed of a 1 cm cuvette, ITO as a working electrode, a platinum wire as an auxiliary electrode, and an Ag/AgCl reference electrode. Absorption spectra in the spectroelectrochemical experiments were measured with an Agilent 8454 UV-Visible diode array spectrophotometer (Santa Clara, CA, USA). Color coordinates of the electrochromic films were measured on an Admesy Brontes colorimeter. Photoluminescence (PL) spectra were measured with a Horiba FluoroMax-4 Spectrofluorometer (Kyoto, Japan). Fluorescent quantum yields (Φ_PL_) of the samples in different solvents were calculated by comparing emission with that of a standard solution of 9,10-diphenylanthracene in cyclohexane (Φ_PL_ = 90%) at room temperature.

### 2.4. Fabrication of Electrochromic Devices

Electrochromic polymer films were electrodeposited on the ITO-coated glass substrate by the electropolymerization method described above. A gel electrolyte based on PMMA (Mw: 120,000) and LiClO_4_ was plasticized with propylene carbonate (PC) to form a highly transparent and conductive gel. PMMA (1 g) was dissolved in dry MeCN (4 mL), and LiClO_4_ (0.1 g) was added to the polymer solution as a supporting electrolyte. Then, propylene carbonate (1.5 g) was added as a plasticizer. The mixture was then gently stirred until gelation. The gel electrolyte was spread on the polymer-coated side of the electrode, and the electrodes were sandwiched.

## 3. Results and Discussion

### 3.1. Synthesis and Structural Characterization

**Scheme 1:**
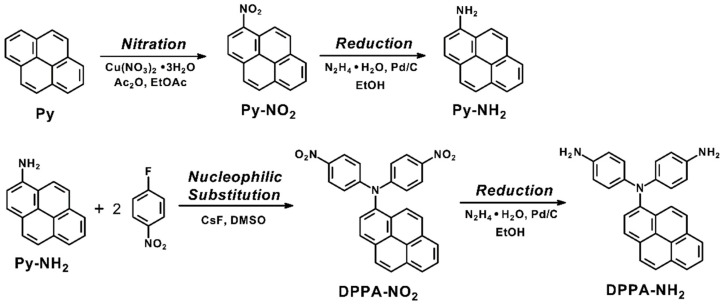
Synthetic routes to 1-aminopyrene (Py-NH_2_) and *N*,*N*-di(4-aminophenyl)-1-aminopyrene (DPPA-2NH_2_).

Due to the highly fluorescent property of pyrene moiety and redox-activity of triarylamine unit, incorporation of diphenylpyrenylamine (DPPA) subunit into the SNS monomer may lead to new compounds and PSNS with dual fluorescent and electrochromic functions. According to Scheme 1, 1-aminopyrene (Py-NH_2_) was synthesized starting from the nitration of pyrene, followed by Pd/C-catalyzed hydrazine reduction of the intermediate 1-nitropyrene (Py-NO_2_). *N*,*N*-Di(4-aminophenyl)-1-aminopyrene (DPPA-2NH_2_) was prepared by CsF-assisted *N*,*N*-diarylation reaction of 1-aminopyrene with *p*-fluoronitrobenzene, followed by Pd/C-catalyzed reduction of the intermediate dinitro compound *N*,*N*-di(4-nitrophenyl)-1-aminopyrene (DPPA-2NO_2_). The synthetic details and characterization data of the synthesized compounds were reported in our previous publication [24]. The IR and ^1^H-NMR spectra of DPPA-2NH_2_ are demonstrated in the Supplementary Materials.

**Scheme 2:**
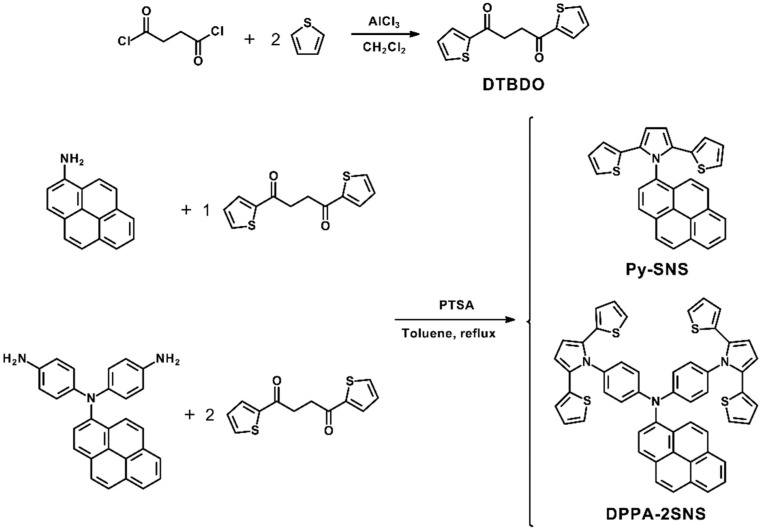
Synthesis of 1,4-di(thiophene-2-yl)butane-1,4-dione (DTBDO), Py-SNS, and DPPA-2SNS.

The SNS-based monomers Py-SNS and DPPA-2SNS were prepared by the Paal–Knorr pyrrole synthesis from Py-NH_2_ and DPPA-2NH_2_, respectively, with 1,4-di(2-thienyl)butane-1,4-dione, using *p*-toluenesulfonic acid (PTSA) as an acid catalyst. The synthetic route is shown in Scheme 2, and all the target compounds were characterized by FTIR and ^1^H NMR spectroscopy.

Figure S1 (Supplementary Materials) illustrates the FT-IR spectra of Py-NH_2_, DPPA-2NH_2_ and their nitro precursors. 1-Nitropyrene and *N*,*N*-di(4-nitrophenyl)-1-aminopyrene show the characteristic nitro absorption pair at 1586/1331 and 1577/1304 cm^−1^ (−NO_2_ asymmetric and symmetric stretching), respectively. After reduction, the characteristic absorptions of the nitro group disappear and the amino compounds show the typical −NH_2_ stretching absorptions in the region from 3200 to 3430 cm^−1^. IR spectra of 1,4-di(2-thienyl)butane-1,4-dione (DTBDO), Py-SNS, and DPPA-2SNS are summarized in Figure S2. Characteristic absorptions agree well with the desired molecular structures. The complete conversion to the N-substituted pyrrole unit could be confirmed by the disappearance of carbonyl absorption at 1565 cm^−1^ and aliphatic C–H stretching absorption at 2922 cm^−1^ of DTBDO and the primary amine absorption in the range of 3200−3430 cm^−1^. The ^1^H NMR spectra of DPPA-2NH_2_, DTBDO, and the SNS monomers Py-SNS and DPPA-2SNS are illustrated in Figures S3–S7. All the resonance peaks can be well assigned to the molecular structures of the synthesized compounds. Although the resonance signals of the pyrenyl protons are somewhat complicated, full assignments of all peaks can be done with the aid of two-dimensional H-H COSY NMR spectra. Thus, the results of all the spectroscopic analysis suggest the successful preparation of the targeted compounds.

### 3.2. UV-Vis Absorption and Photoluminescence of DPPA-2SNS Monomer

**Figure 1:**
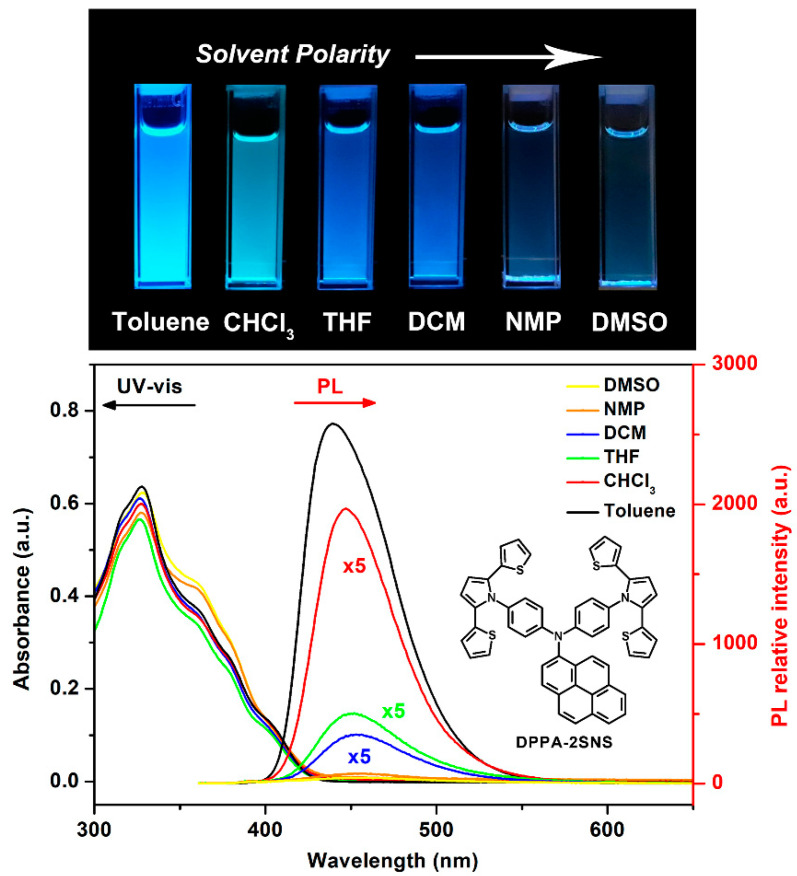
PL images, UV-vis absorption, and PL profiles of the dilute solutions (ca. 1 × 10^−5^ M) of DPPA-2SNS in various solvents. Photographs were taken under illumination of 365 nm UV light.

UV-Vis absorption and photoluminescence (PL) spectra of DPPA-2SNS in dilute solution (~1 × 10^−5^ M) and the PL image of the solution under 365 nm light are shown in Figure 1. Their absorption and PL data are summarized in Table S1. The solutions of DPPA-2SNS in different solvents show absorption maxima in the range of 326~328 nm. The absorption maximum shows little shift in the tested solvents, indicating that the solvent polarity exerts little effect on its ground-state electronic transition. DPPA-2SNS is fluorescent and exhibits a PL maximum wavelength of 439 nm, resulting in blue emission in toluene solution. The PL emission of DPPA-2SNS displays a slight solvent-polarity dependence, revealing a dominant broad emission band that undergoes remarkable bathochromic shifts with an increase in the solvent polarity. The emission wavelength slightly increased with increasing solvent polarity, changing from PL λ_max_ of 439 nm in toluene to 453 nm in DMSO. The quantum yield of DPPA-2SNS diminished significantly from 35% in toluene to 0.2% in DMSO. The fluorescence solvatochromism can be attributed to the fast intramolecular charge-transfer process, resulting in a large change in dipole moment in the excited state.

### 3.3. Electrochemical Activity and Polymerization of SNS Monomers

**Figure 2:**
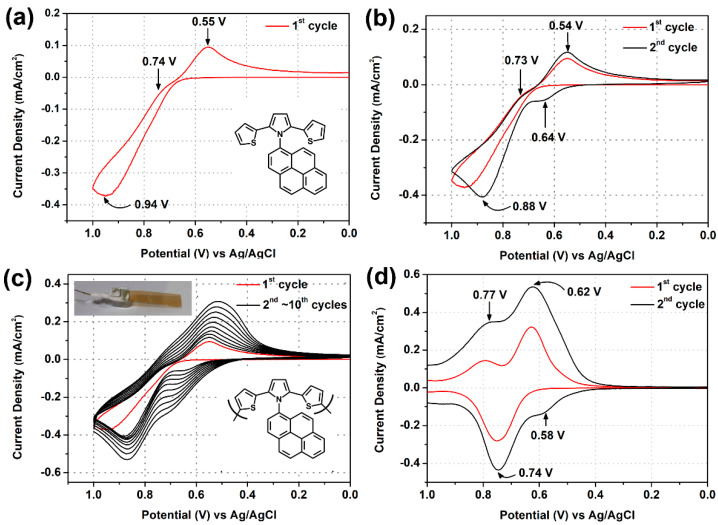
(**a**) The first CV scan, (**b**) the first two CV scans, and (**c**) ten repetitive CV scans of 1 mM of Py-SNS in 0.1 M TBAP/MeCN in the potential range of 0.00–1.00 V at a scan rate of 50 mV/s. (**d**): DPV scans at scan rate of 5 mV/s.

The electrochemical properties of Py-SNS and DPPA-2SNS2 were probed by cyclic voltammetry in 0.1 M Bu_4_NClO_4_ solution of acetonitrile (MeCN) or dichloromethane (CH_2_Cl_2_). Figure 2 presents the cyclic voltammetry (CV) diagrams of 1 mM Py-SNS in 0.1 M Bu_4_NClO_4_/MeCN at a scan rate of 50 mV/s between 0.00 and 1.00 V. As shown in the first CV scan, the SNS unit started oxidation at about 0.7 V, followed by an oxidation peak at 0.94 V. In the second scan, a new oxidation peak appeared at around 0.64 V and intensified after each cycle, which indicated the occurrence of the coupling reactions between terminal thiophene cation radicals forming the bis- or oligo-SNS moiety. After 10 cycles of CV scan, a perceived yellowish orange polymer film was deposited on the ITO-glass (as shown in the inset of Figure 2c). The polymer film adhered firmly on the electrode surface and could not be removed from the ITO-glass substrate, even after being immersed in water for a month; however, the film could completely dissolve in NMP and concentrated sulfuric acid under ultrasonic oscillation in two hours.

Figure S8 shows the CV diagrams of 1 mM Py-SNS in 0.1 M Bu_4_NClO_4_/CH_2_Cl_2_ at a scan rate of 50 mV/s between 0.00 and 1.10 V. Similar to that observed in acetonitrile, a new oxidation peak at 0.66 V was observed in the second CV scan; however, the current density of the redox waves did not increase with successive scans, and the monomer did not give the corresponding polymer on the electrode surface in dichloromethane. The result is similar to the finding reported in the literature [46]. Thus, the electropolymerization of monomer Py-SNS was carried out in 0.1 M Bu_4_NClO_4_/MeCN.

**Figure 3:**
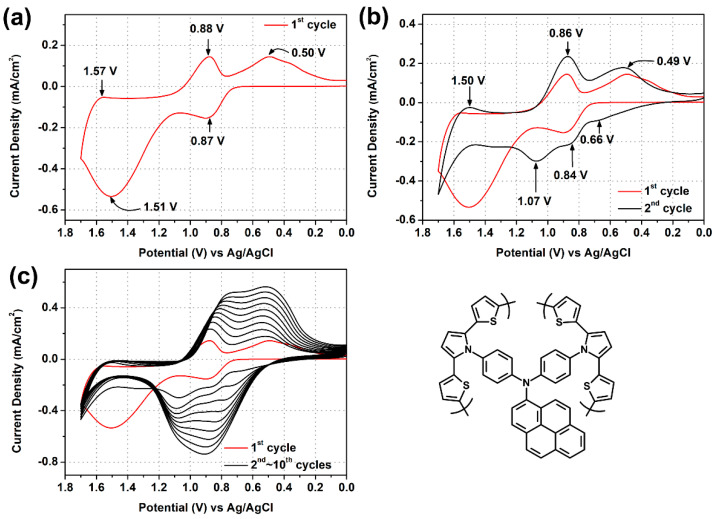
(**a**) The first CV scan, (**b**) the first two second CV scan, and (**c**) ten repetitive CV scans of 0.2 mM of DPPA-2SNS in 0.1 M Bu_4_NClO_4_/CH_2_Cl_2_ in the potential range of 0.00–1.70 V at a scan rate of 50 mV/s.

DPPA-2SNS is hardly soluble in acetonitrile, but it is readily soluble in dichloromethane. Therefore, the CV experiments of this SNS monomer were performed in dichloromethane. Figure 3 illustrates the CV diagrams of DPPA-2SNS (0.2 mM) in a 0.1 M Bu_4_NClO_4_/CH_2_Cl_2_ solution over a range of applied potentials of 0.00–1.70 V. There are two oxidation waves at around 0.87 and 1.51 V observed on the first CV cycle, which can be attributed to the oxidation of the SNS moiety and the combined oxidation reactions of the amino center of DPPA and the pyrene group, respectively. Due to the in situ coupling reaction of the SNS units during the first CV cycle, a new redox couple appeared at 0.66 and 0.49 V in the subsequent cycle. The oxidation peak at 1.07 V in the second CV cycle should be related to the oxidation of the DPPA amino center. In addition, the gradual broadening of the redox waves could be clearly seen in successive scans, indicating the gradual deposition of electroactive species on the working electrode. After ten CV scans, a visible dark blue polymer film formed on the ITO electrode. The electrodeposited film was insoluble in NMP and concentrated sulfuric acid because of the crosslinking structure of the polymer matrix. The polymer film can be stripped off from the ITO-glass after being immersed in water.

**Figure 4:**
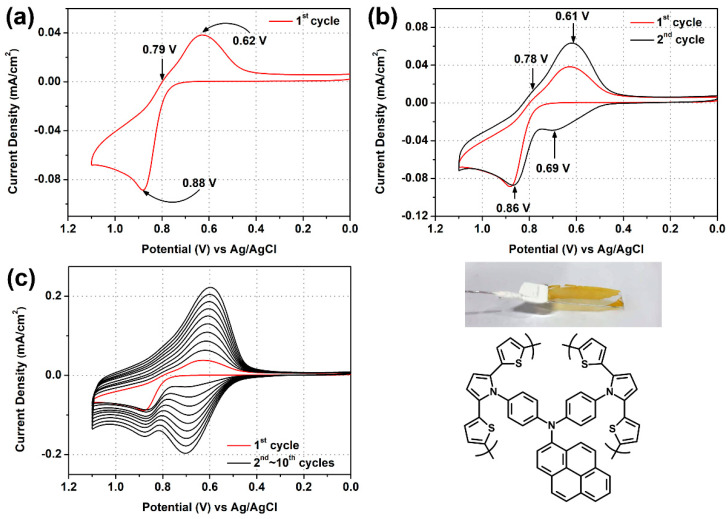
(**a**) The first CV scan, (**b**) the first two second CV scan, and (**c**) ten repetitive CV scans of 0.2 mM of DPPA-2SNS in 0.1 M Bu_4_NClO_4_/CH_2_Cl_2_ in the potential range of 0.00–1.10 V at a scan rate of 50 mV/s.

**Figure 5:**
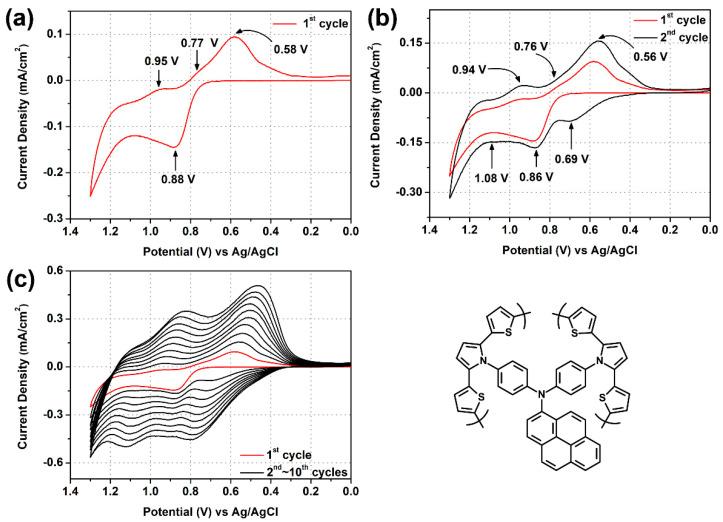
(**a**) The first CV scan, (**b**) the first two second CV scan, and (**c**) ten repetitive CV scans of 0.2 mM of DPPA-2SNS in 0.1 M Bu_4_NClO_4_/CH_2_Cl_2_ in the potential range of 0.00–1.30 V at a scan rate of 50 mV/s.

However, the applied voltage of 1.7 V may cause an over-oxidation of the deposited polymer. As shown in Figure 4 and Figure 5, monomer DPPA-2SNS could undergo electrochemical polymerization by repeated CV scanning in a range of applied potentials of 0.00–1.10 or 0.00–1.30 V.

### 3.4. UV-vis Absorption of the PSNS Films

**Figure 6:**
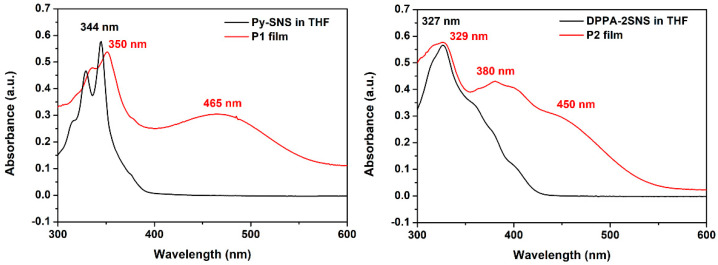
UV-vis absorption spectra of Py-SNS and DPPA-2SNS in THF (ca. 1 × 10^−5^ M) and their electrodeposited films on ITO-glass.

The UV-vis absorption spectra of the solutions of monomers Py-SNS (in MeCN) and DPPA-2SNS (in CH_2_Cl_2_) and their corresponding polymer films of **P1** and **P2** on an ITO-glass substrate are depicted in Figure 6. The spectra of the monomers show absorption bands with maximum peaks at 342 and 326 nm and absorption onsets at 390 and 428 nm, respectively. The polymer films of **P1** and **P2** show absorption maxima at 350 and 329 nm and absorption onsets at 569 and 547 nm, respectively. The red-shift in absorption maximum and onset of the polymer films compared to the monomers imply an extended π-conjugation length. As mentioned earlier, the dilute solutions of DPPA-2SNS in less polar solvents such as toluene showed a moderate fluorescence intensity. However, the electrodeposited film of **P2** revealed a very weak fluorescence emission, possibly due to a high degree of π-π stacking of the aromatic units in the solid state.

### 3.5. Redox Response of Polymers

**Figure 7:**
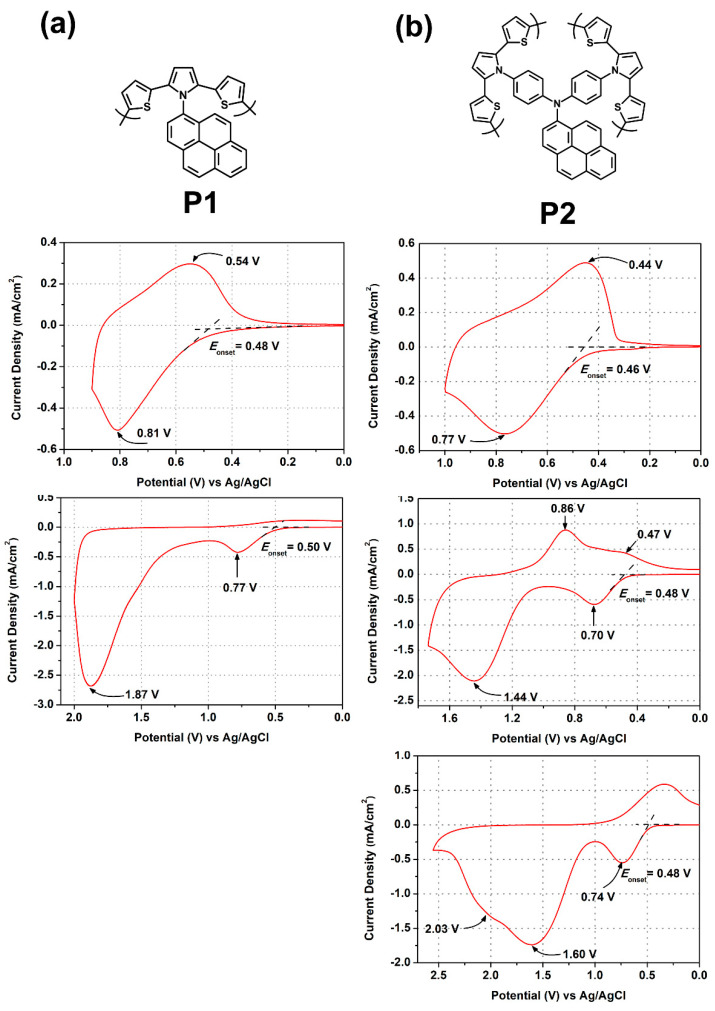
Cyclic voltammorgrams of the electrodeposited films of (**a**) **P1** from Py-SNS and (**b**) **P2** from DPPA-2SNS on the ITO-coated glass slide in 0.1 M Bu_4_NClO_4_/MeCN at a scan rate of 50 mV/s.

The electrochemical properties of the polymer films **P1** and **P2** electrodeposited on ITO glass were investigated by CV in a 0.1 M Bu_4_NClO_4_/MeCN monomer-free solution. As illustrated in Figure 7a, the **P1** film electropolymerized from Py-SNS exhibited one reversible redox couple (*E*_1/2_ = 0.68 V) with *E*_pa_ = 0.81 V and *E*_pc_ = 0.54 V when the applied potential was scanned between 0 and 0.9 V. The reversible oxidation process should be associated with the oxidation of the SNS units in the polymer chain. When the potential was scanned to 2.0 V, two oxidation waves were observed at 0.77 and 1.87 V. However, the oxidation processes were irreversible due to over-oxidation of the polymer. Similarly, the CV diagram of the **P2** film from DPPA-2SNS showed one reversible oxidation process (*E*_1/2_ = 0.61 V, *E*_pa_ = 0.77 V and *E*_pc_ = 0.44 V) in the potential range of 0.0–1.0 V, attributed to the oxidation reaction of the SNS units (see Figure 7b). Two semi-reversible oxidation processes with oxidation peaks at 0.70 and 1.44 V were observed in the CV diagram with the scanning range of 0.0–1.7 V. As the potential was scanned up to 2.5 V, **P2** showed two or three oxidation waves. The first oxidation process is reversible; however, the latter oxidation processes are irreversible, probably due to over-oxidation of the polymer.

Figure S9 compares the CV behaviors of two polymer films of **P2** prepared by the different potential scanning ranges of 0.0–1.1 V (film A) and 0.0–1.3 V (film B) for ten repetitive CV scans. These two films show similar CV behaviors, as the voltage was applied between 0 and 1.2 or 1.3 V. Two redox pairs were observed in their CV diagrams, which could be ascribed to the oxidation reactions of the SNS moiety and the DPPA amino center, respectively. As can be seen from the CV diagrams cycled in the 0.0–1.7 V range, the polymer film A seems to reveal a higher electrochemical activity of the DPPA core (*E*_pa_ at around 0.95 V) than polymer film B. Thus, the polymer **P2** from DPPA-2SNS prepared by repeated CV scanning in the applied potential range of 0.0–1.1 V was expected to possess better electrochromic performance. Redox waves at lower potentials of these two polymers are apparently attributable to reversible electrochemical oxidation processes of the SNS units, and the second redox wave at around 0.94 V in the CV diagram of **P2** is ascribed to the oxidation of amino center in the DPPA core.

The energy levels of the highest occupied molecular orbital (HOMO) and lowest unoccupied molecular orbital (LUMO) of the corresponding polymers were estimated from the oxidation *E*_onset_ values (Table 1). Assuming that the HOMO energy level for the ferrocene/ferrocenium (Fc/Fc^+^) standard is 4.80 eV with respect to the zero vacuum level, the HOMO levels for **P1** and **P2** were calculated to be 4.84 and 4.83 eV (relative to the vacuum energy level), respectively. Their LUMO energy levels were estimated to be −2.66 and −2.56 eV, respectively, deduced from the bandgap calculated from the optical absorption edge. Figure S10 displays the CV curves of the polymer films at different scanning rates between 30 and 300 mV/s in 0.1 M Bu_4_NClO_4_/MeCN, and the redox current density presents a linear growth with the increasing scan rate. This indicates that the polymer films were firmly coated on the ITO electrode and they showed a non-diffusional redox behavior.

**Figure 8:**
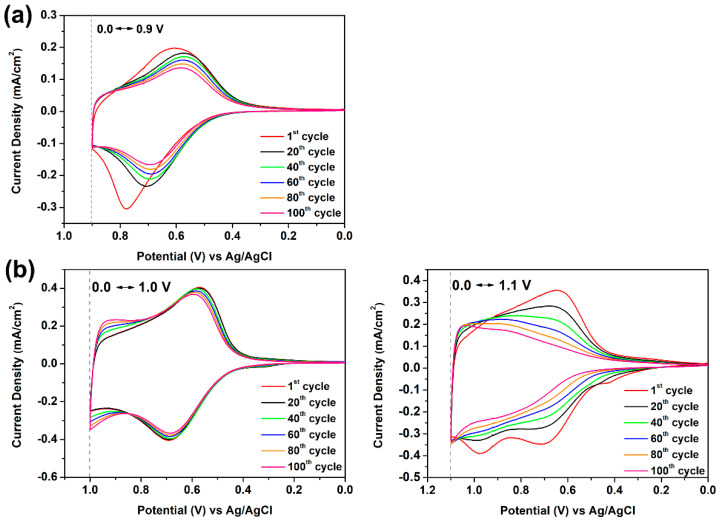
CV diagrams of (**a**) **P1** and (**b**) **P2** on the ITO-coated glass slide in 0.1 M Bu_4_NClO_4_/MeCN at a scan rate of 50 mV/s.

**Figure 9:**
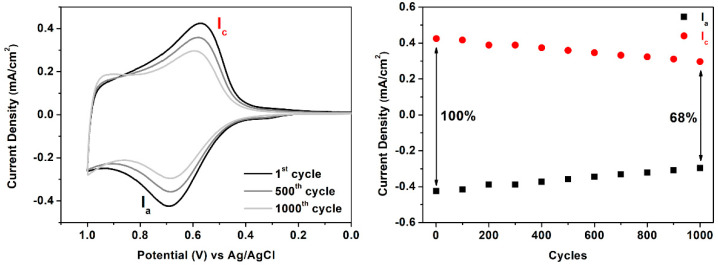
Electrochemical stability of **P2** on the ITO-coated glass slide in 0.1 M Bu_4_NClO_4_/MeCN after 1000 repetitive switching by a CV method with scan rate at 50 mV/s. I_a_: anodic peak current, I_c_: cathodic peak current.

All the polymer films still remained a very high redox-activity after 100 repetitive CV sans in the potential ranges of 0.0–0.9 V or 0.0–1.0 V (see Figure 8), especially with electron-rich triarylamine unit, indicating high electrochemical stability in the first oxidized state. However, the second oxidized state of polymer **P2** is less stable as seen from the decrease of redox current density upon long-term CV cycling. For commercial electrochromic application, long-term cycling stability is a key issue for materials and their derived devices. Figure 9 shows that the **P2** film has a reasonable stability, since it retains 68% of its electroactivity after 1000 repetitive switches between neutral state and first oxidized states.

### 3.6. Spectroelectrochemical Properties of Polymers

**Figure 10:**
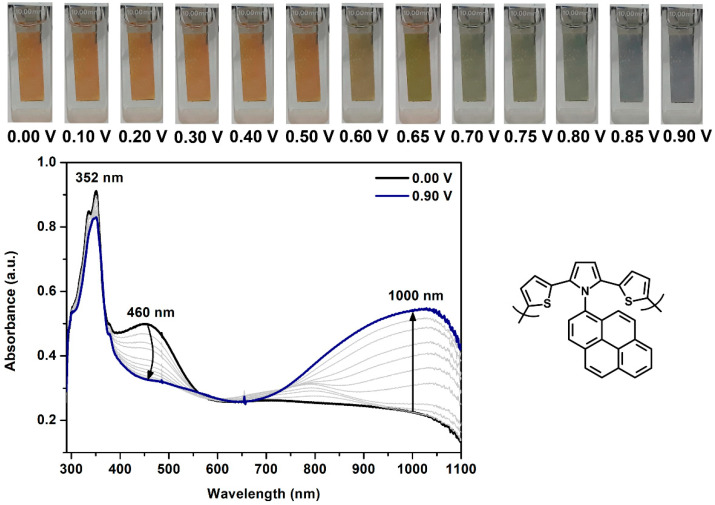
Spectroelectrograms, color changes and transmittance changes of **P1** thin films on ITO-coated glass in 0.1 M Bu_4_NClO_4_/MeCN at various applied voltages.

The spectroelectrochemical properties of the electrodeposited polymer films were examined for various redox states by using the changes in the electronic absorption spectra under various applied potentials. In the neutral form, the **P1** film exhibited strong absorption in the UV region at wavelength 352 nm and a medium absorption in the visible region at 460 nm; thus, the film appeared as yellowish orange in color (Figure 10). When the voltage was gradually raised to 0.90 V, the absorption intensity at 460 nm dropped obviously and a new broad band appeared in the near-infrared (NIR) region centered at 1000 nm, corresponding to the formation of the charge carriers. During oxidation, the polymer film induced a color change from yellowish orange (*L* * = 46, *a* * = 11, *b* * = 26) to blue (*L* * = 39, *a* * = 0, *b* * = −1).

**Figure 11:**
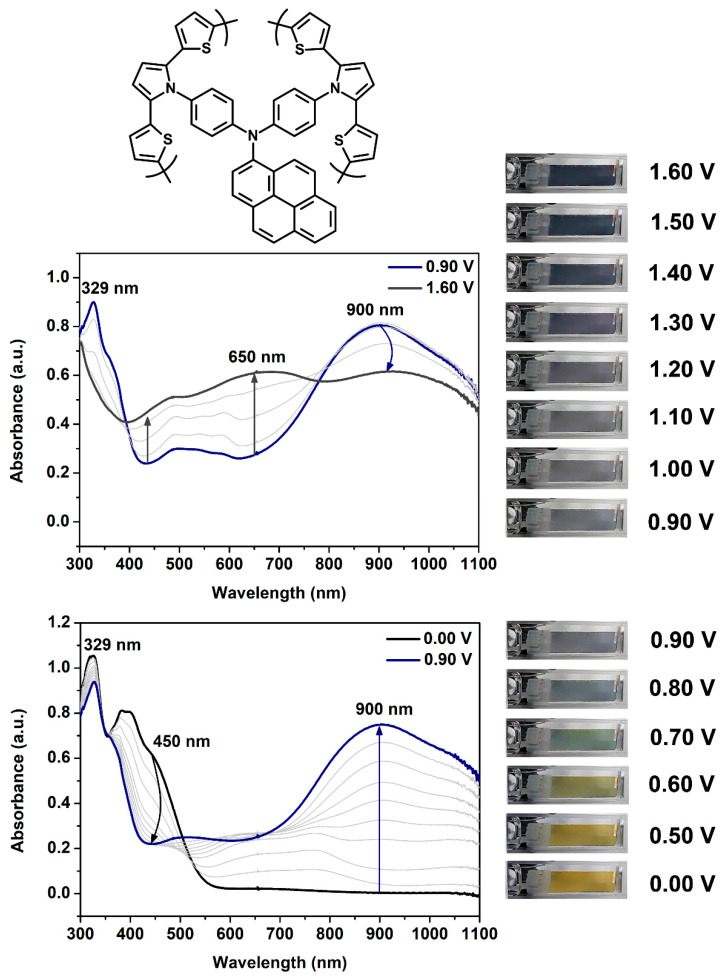
Spectroelectrograms and color changes of **P2** thin films on ITO-coated glass in 0.1 M Bu_4_NClO_4_/MeCN at various applied voltages.

The spectral changes in the **P2** film electro-deposited from DPPA-2SNS correlated with applied potentials are presented in Figure 11. **P2** revealed a strong UV absorption at 329 nm and a medium absorption in the visible region of 400–500 nm in the neutral state and appeared as yellowish orange (*L* * = 54, *a* * = 2, *b* * = 40). As the voltage was slowly increased from 0.00 to 0.90 V, the absorbance at 329 and 450 nm diminished, and a broad absorption in the near infrared (NIR) region emerged at around 900 nm, accompanied by a color change of the film to greyish blue (*L* * = 52, *a* * = 2, *b* * = −7). The spectra and color change of the **P2** film can be explained by the formation of polaron charge carriers caused by oxidation of the SNS units. As the applied voltage was raised to 1.60 V, the NIR absorbance slightly dropped, and the growth of new broad band in the visible region of 400–800 nm was observed, which indicated the formation of bipolaron charge carriers caused by further oxidations of the amino center of DPPA group and the SNS moiety; meanwhile, the **P2** film changed color from greyish blue to purplish black (*L* * = 14, *a* * = 3, *b* * = −5).

### 3.7. Electrochromic Switching

Electrochromic switching studies were carried out in order to elucidate the response times and optical contrast of the polymer films. The absorbance of the polymer films at an absorption maximum was monitored as a function of time when the film was switched between neutral and oxidized states.

Figure S11 depicts the optical transmittance of **P1** film at 460 and 1000 nm as a function of time by applying square-wave potential steps between 0 and 0.90 V for a pulse width of 10 s. The response time was calculated as the time required 90% of full switches between their colored and bleached stages because after this point the naked eyes could not sense the changes in the color. The optical contrast (measured as Δ%T) of **P1** between neutral orange and oxidized blue states was found to be 16% at 460 nm and 32% at 1000 nm. The response time for the coloring and bleaching process is about 3 and 1 s, respectively.

**Figure 12:**
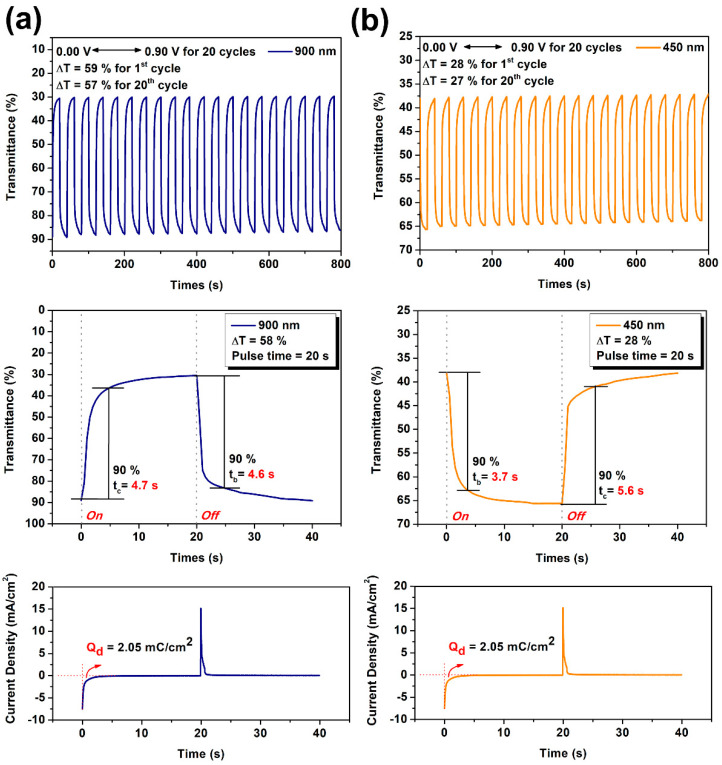
Potential step absorptiometry of the **P2** film (from DPPA-2SNS) on the ITO-glass slide (in MeCN with 0.1 M Bu_4_NClO_4_ as a supporting electrolyte) by applying a potential step: (**a**) 0.00 V ↹ 0.90 V (20 cycles) with a pulse width of 20 s at λ_max_ = 900 nm and (**b**) 0.00 V ↹ 0.90 V (20 cycles) with a pulse width of 20 s at λ_max_ = 450 nm. The optical contrast and response times were calculated for the first switching cycle.

When the **P2** film was switched between 0.00 and 0.90 V with a pulse width of 20 s, the optical transmittance at 450 and 900 nm was examined. As shown in Figure 12, the optical contrast of **P2** between neutral yellow and oxidized greyish blue states was found to be 28% at 450 nm and 58% at 900 nm. The coloring response time was measured as 3.7 s at 450 nm and 4.7 s at 900 nm, and the bleaching response time was recorded as 5.6 s at 450 nm and 4.6 s at 900 nm. As the applied voltage was stepped from 0.00 to 1.60 V, the film exhibited optical contrast of 29% at 650 nm for the oxidized purplish black state and required 8.1 s for the coloring step and 15.3 s for the bleaching step, as shown in (Figure S12). However, the **P2** film revealed a substantial loss of optical contrast after 10 switching cycles between the neutral and the second oxidized states (from 29% to 13%).

The electrochromic coloration efficiency (CE) can be calculated by the equation: CE = ΔOD/Q_d_, where ΔOD is the optical absorbance change, and Q_d_ is the inject/ejected charge during redox step. As can be seen from Table 2, the CE values of **P1** were measured as 108 cm^2^/C at 1000 nm and 62 cm^2^/C at 460 nm. Those of **P2** were calculated as 224 cm^2^/C at 900 nm and 117 cm^2^/C at 450 nm.

As mentioned earlier, polymer **P2** seemed to exhibit a longer response time than polymer **P1**. The most probable reason might be that the cross-linked structure of **P2** is not conducive to countering ion (TBA^+^) penetration. Therefore, the electrolyte was changed to lithium perchlorate (LiClO_4_) with a smaller counter ion Li^+^. As shown in Figure S13 and Table S2, a faster response time was observed when the switching test of the **P2** film was carried out in a LiClO_4_/MeCN solution.

### 3.8. Electrochromic Devices

**Figure 13:**
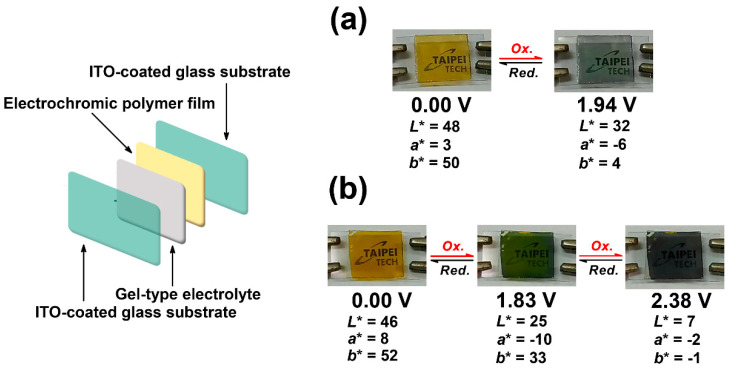
Photos of sandwich-type ITO-coated glass electrochromic devices using (**a**) **P1** and (**b**) **P2** films as active layers (active area ca. 20 mm × 20 mm).

Based on the foregoing results, it can be concluded that the electrochemically generated polymer films can be used in the construction of electrochromic devices and the optical display. Therefore, we fabricated single-layer electrochromic cells as preliminary investigations. The polymer films were electrodeposited onto ITO-coated glass, thoroughly rinsed, and then dried. Afterward, the transparent and conductive gel electrolyte was spread on the polymer-coated side of the electrode and the electrodes were sandwiched. To prevent leakage, an epoxy resin was applied to seal the device. The electrochromic devices using **P1** and **P2** films as active layers were fabricated (Figure 13). The **P1**-based device is pale yellowish orange in the neutral form. When the applied voltage was increased to 1.94 V, the color changed to greenish blue. In the other case, the **P2**-based device is yellowish orange in the neutral form. When the applied voltage was increased to 1.83 and 2.38 V, the color changed to greenish blue and purplish black, respectively.

## 4. Conclusions

In this work, a new SNS-based electroactive molecule DPPA-2SNS, bearing triarylamine subunits, was synthesized and then directly deposited onto the ITO-glass substrate as a robust polymeric film by repetitive CV scanning. The CV diagrams of the electrochemically generated polymers revealed multiple oxidation processes, and the first two oxidation reactions were reversible. A multi-colored electrochromism with yellowish orange, greyish blue, and purplish black colors was observed in the spectroelectrochemical measurement of the electrodeposited thin films by applying a positive potential. These polymers also showed strong near-infrared absorption upon oxidation. The polymer films generally exhibited reasonable coloration efficiency and optical contrast, together with high switching stability, especially at the first oxidized state. Thus, the prepared polymers can be promising candidates for electrochromic applications.

## Figures and Tables

**Table 1:** Optical and electrochemical properties of the electrosynthesized polymers.

Polymers	Structures	UV-Vis Absorption (nm) ^a^		Oxidation Potential (V) ^b^			Optical Bandgap (*E*_g_) (eV) ^c^	HOMO (eV) ^d^	LUMO (eV) ^d^
		λ_max_	λ_onset_	*E*_onset_	*E*_1/2_^Ox1^	*E*_1/2_^Ox2^			
**P1**	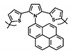	350	569	0.48	0.68	–	2.18	−4.84	−2.66
**P2**	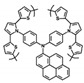	329	547	0.47	0.59	1.15	2.27	−4.83	−2.56

Notes: ^a^ UV-vis absorption maximum and onset wavelengths for the polymer films; ^b^ Calculated from first CV scans, versus Ag/AgCl in MeCN at a scan rate of 50 mV/s; ^c^ Optical band gap calculated from absorption edge of the polymer film: *E*_g_ = 1240/λ_onset_; ^d^ The HOMO and LUMO energy levels were calculated from *E*_onset_ values of CV diagrams and were referenced to ferrocene (4.8 eV relative to vacuum energy level; *E*_onset_ = 0.44 V; *E*_1/2_ = 0.52 V in CH_2_Cl_2_). −*E*_HOMO_ = *E*_onset_ + 4.8 − 0.44 (eV); −*E*_LUMO_ = –*E*_HOMO_ − *E*_g_.

**Table 2:** Electrochromic properties of the polymer films.

Polymers	λ_max_^a^ (nm)	Δ%T	Response Time ^b^		ΔOD ^c^	Q_d_^d^ (mC/cm^2^)	CE ^e^ (cm^2^/C)
			t_c_ (s)	t_b_ (s)			
**P1**	1000	32	3.3	1.0	0.30	2.76	108
	460	16	1.3	2.9	0.17	2.76	62
**P2**	900	58	4.7	4.6	0.46	2.05	224
	450	28	5.6	3.7	0.24	2.05	117
	650	45	8.1	15.3	0.59	9.09	65

Notes: ^a^ Wavelength of absorption maximum; ^b^ Time for 90% of the full-transmittance change; ^c^ Optical density change (ΔOD) = log[T_bleached_/T_colored_], where T_colored_ and T_bleached_ are the maximum transmittance in the oxidized and neutral states, respectively; ^d^ Q_d_ is ejected charge, determined from the in situ experiments; ^e^ Coloration efficiency (CE) = ΔOD/Q_d_.

## Supplementary Materials

The following are available online at https://www.mdpi.com/2073-4360/12/12/2777/s1, Figure S1: IR spectra of the 1-nitropyrene, 1-aminopyrene, *N*,*N*-di(4-nitrophenyl)-1-aminopyrene, and *N*,*N*-di(4-aminophenyl)-1-aminopyrene. Figure S2: IR spectra of the 1,4-di(thiophen-2-yl)butane-1,4-dione, Py-SNS, and DPPA-2SNS. Figure S3: ^1^H NMR spectrum of *N*,*N*-di(4-aminophenyl)-1-aminopyrene in DMSO-*d*_6_. Figure S4: ^1^H NMR spectrum of 1,4-di(thiophen-2-yl)butane-1,4-dione in CDCl_3_ (* solvent peak). Figure S5: ^1^H NMR and H-H COSY spectra of Py-SNS in DMSO-*d*_6_. Figure S6: ^1^H NMR spectrum of DPPA-2SNS in CDCl_3_ (* solvent peak). Figure S7: H-H COSY spectra of DPPA-2SNS in CDCl_3_ (* solvent peak). Figure S8: (a) The first CV scan, (b) the first two second CV scan, and (c) ten repetitive CV scans of 1 mM of Py-SNS in 0.1 M Bu_4_NClO_4_/CH_2_Cl_2_ in the potential range of 0.00–1.10 V at a scan rate of 50 mV/s. Figure S9: Cyclic voltammorgrams of the electrodeposited films of DPPA-2SNS on the ITO-coated glass slide in 0.1 M Bu_4_NClO_4_/MeCN at a scan rate of 50 mV/s. (a) Film A prepared by repeated CV scanning between 0 and 1.1 V and (b) film B prepared by repeated CV scanning between 0 and 1.3 V for ten cycles. Figure S10: Scan rate dependence of polymer films (a) P1 and (b) P2 on the ITO-glass substrate in MeCN containing 0.1 M Bu_4_NClO_4_ at different scan rates from 30 to 300 mV/s. Figure S11: Potential step absorptiometry of the P1 film (from Py-SNS) on the ITO-glass slide (in MeCN with 0.1 M Bu_4_NClO_4_ as a supporting electrolyte) by applying a potential step: (a) 0.00 V ↹ 0.90 V (20 cycles) with a pulse width of 10 s at λ_max_ = 1000 nm and (b) optical switching at potential 0.00 V ↹ 0.90 V (20 cycles) with a pulse width of 10 s at λ_max_ = 460 nm. The optical contrast and response times were calculated for the first switching cycle. Figure S12: Potential step absorptiometry of the P2 film (from DPPA-2SNS) on the ITO-glass slide (in MeCN with 0.1 M Bu_4_NClO_4_ as a supporting electrolyte) by applying a potential step 0.00 V ↹ 1.60 V (10 cycles) with a pulse width of 30 s at λ_max_ = 650 nm. The optical contrast and response times were calculated for the first switching cycle. Figure S13: Potential step absorptiometry of the P2 film (from DPPA-2SNS) on the ITO-glass slide (in MeCN with 0.1 M LiClO_4_ as a supporting electrolyte) by applying a potential step: (a) 0.00 V ↹ 0.90 V (20 cycles) with a pulse width of 15 s at λ_max_ = 900 nm and (b) 0.00 V ↹ 0.90 V (20 cycles) with a pulse width of 15 s at λ_max_ = 450 nm. The optical contrast and response times were calculated for the first switching cycle. Table S1: Optical properties of DPPA-2SNS in different solvents. Table S2: Electrochromic properties of the polymer films of P2 with different electrolyte.
