# Supplementary material for: Electrosynthesis and Electrochromism of a New Crosslinked Polydithienylpyrrole with Diphenylpyrenylamine Subunits
Source: Polymers (Basel). 2020 Nov 24;12(12):2777. doi: 10.3390/polym12122777 (PMC7760582; doi:10.3390/polym12122777)
Supplement: Supplementary file 1 [file polymers-12-02777-s001.pdf]

Supplementary Materials

Electrosynthesis and electrochromism of a new crosslinked polydithienylpyrrole with diphenylpyrenylamine subunits

Yu-Ruei Kung[a,*], Sin-Yun Cao[b] and Sheng-Huei Hsiao[b,*]

[a] *Department of Chemical Engineering and Biotechnology, Tatung University, Taipei, Taiwan*

[b] *Department of Chemical Engineering and Biotechnology, National Taipei University of Technology, Taipei, Taiwan*

* Corresponding authors.

E-mail address: yrkung@gm.ttu.edu.tw (Y.-R. Kung), shhsiao@ntut.edu.tw (S.-H. Hsiao)

1. Experimental section

1.1 Materials

Succinyl chloride (TCI), thiophene (Acros), aluminum (Ⅲ) chloride ($AlCl_3$) (TCI), *p*-toluenesulfonic acid monohydrate (PTSA) (TCI) were used as received. Toluene (Echo) and dichloromethane (Alfa Aesar) were dried over calcium hydride for 24 h, and then distilled and stored over 4 Å molecular sieves in a sealed bottle. Pyrene (Acros), *p*-anisidine (Acros), 4-fluoronitrobenzene (Alfa Aesar), copper (Ⅱ) nitrate trihydrate (Showa), acetic anhydride (Tedia), cesium fluoride (CsF) (Acros), hydrazine monohydrate (Alfa Aesar), 10% palladium on charcoal (Pd/C) (Lancaster), dimethyl sulfoxide (DMSO) (Macron) were also used as received from commercial sources.

1-Aminopyrene was synthesized starting from the nitration of pyrene, followed by Pd/C-catalyzed hydrazine reduction of the intermediate 1-

nitropyrene. *N,N*-Di(4-aminophenyl)-1-aminopyrene (DPPA-2NH_2) was prepared by CsF-assisted *N,N*-diarylation reaction of 1-aminopyrene with *p*-fluoronitrobenzene, followed by Pd/C-catalyzed reduction of the intermediate dinitro compound *N,N*-di(4-nitrophenyl)-1-aminopyrene. The synthetic details and characterization data of the synthesized compounds have been reported previously [S1]*.

*S1 Y.-C. Kung, S.-H. Hsiao, Fluorescent and electrochromic polyamides with pyrenylamine chromophore, *J. Mater. Chem.* **2010**, *20*, 5481–5492.

1.2. 1,4-Di(2-thienyl)butane-1,4-dione (DTBDO)

DTBDO was synthesized by the Friedel-Crafts acylation reaction of thiophene with succinyl chloride in the presence of $AlCl_3$. In a 250-mL three-neck round-bottle flask equipped with a stirring bar under nitrogen atmosphere, 8 g (0.06 mol) of $AlCl_3$ was suspended in 130 mL of dichloromethane (CH_2Cl_2). A mixture of 2.75 mL succinyl chloride (0.025 mol) and 4.8 mL thiophene (0.06 mol) in 15 mL CH_2Cl_2 was added dropwise to the suspension solution of $AlCl_3$, and then the mixture was stirred at room temperature for 3.5 h. The resulting reaction mixture was then poured into ice water. After that, concentrated HCl (5 mL) was added and the mixture turned to dark green, which was washed with saturated $NaHCO_3$ solution and dried over $MgSO_4$.The solution was evaporated under reduced pressure to remove most of the solvent, and then the residue was recrystallized by CH_2Cl_2/hexane (1:1) and dried in vacuum at 30 °C to give 5.37 g (86 % in yield) of white crystals with a mp of 130–131 °C (by DSC at a scan rate of 5 °C/min).

FT-IR (KBr): 1513 cm^{-1} (aromatic C=C str.), 1656 cm^{-1} (carbonyl group C=O str.), 2922 cm^{-1} (aliphatic C–H str.), 3107 cm^{-1} (aromatic C–H str.). ^1H NMR (600

MHz, $CDCl_3$, δ, ppm): 3.39 (s, 4H, H_a), 7.14 (t, *J* = 3.8 Hz, 2H, H_c), 7.64 (d, *J* = 3.8 Hz, 2H, H_d), 7.81 (d, *J* = 3.8 Hz, 2H, H_b).

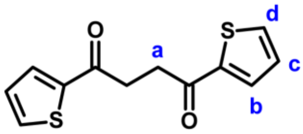

1.3. N-(1-Pyrenyl)-2,5-di(2-thienyl)pyrrole (Py-SNS)

In a 250-mL three-neck round-bottle flask equipped with a stirring bar under nitrogen atmosphere, 0.96 g 1-aminopyrene (**1**) (4 mmol), 1.33 g 1,4-di(2-thienyl)butane-1,4-dione (5.3 mmol) and 0.04 g PTSA (0.2 mmol) were dissolved in 120 mL of toluene. The solution was heated at reflux temperature for 5 days, then evaporated under reduce pressure to remove toluene. The residue was purified by column chromatography eluting with CH_2Cl_2/hexane (1:1), recrystallized from toluene and dried in vacuum at 40 °C to give 0.86 g (50 % in yield) orange crystals with mp = 238–239 °C (by DSC at a heating rate of 5 °C/min).

FT-IR (KBr): 1513 cm^{-1} (aromatic C=C str.), 3107–3034 cm^{-1} (aromatic C–H str.). ^1H NMR (600 MHz, DMSO-d_6, δ, ppm): 6.60 (d, *J* = 3.7 Hz, 2H, H_j), 6.66 (t, *J* = 3.7 Hz, 2H, H_k), 6.83 (s, 2H, H_m), 7.01 (d, *J* = 3.7 Hz, 2H, H_l), 7.32 (d, *J* = 9.1 Hz, 1H, H_a), 8.14 (t, *J* = 7.6 Hz, 1H, H_f), 8.16 (d, *J* = 9.1 Hz, 1H, H_b), 8.21 (d, *J* = 7.9 Hz, 1H, H_i), 8.31 (d, *J* = 7.1 Hz, 1H, H_g), 8.34 (overlapped doublets, 2H, $H_{c,d}$), 8.42 (d, *J* = 7.6 Hz, 1H, H_e), 8.45 (d, *J* = 7.9 Hz, 1H, H_h).

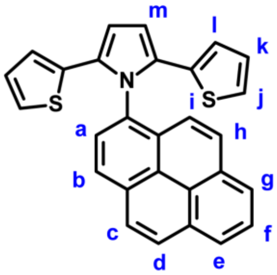

1.4. N,N-Bis(4-(2,5-di(2-thienyl)-1H-pyrrol-1-yl)phenyl)-1-aminopyrene (DPPA-2SNS)

In a 250-mL three-neck round-bottle flask equipped with a stirring bar under nitrogen atmosphere, 1 g *N,N*-di(4-aminophenyl)-1-aminopyrene (**2**) (2.5 mmol), 1.5 g 1,4-di(2-thienyl)butane-1,4-dione (6 mmol) and 0.08 g PTSA (0.42 mmol) were dissolved in 120 mL of toluene. The solution was heated under reflux for 3 days, then evaporated under reduced pressure to remove toluene. The crude product was purified by column chromatography eluting with CH_2Cl_2/hexane (1:1) and dried in vacuum at 30 °C to give 1.13 g (55 % in yield) pale yellow powder with mp = 241–242 °C (by DSC at 5 °C/min).

FT-IR (KBr): 3109–3012 cm^{-1} (aromatic C–H str.), 1513 cm^{-1} (aromatic C=C str.). ^{1}H NMR (600 MHz, $CDCl_3$, δ, ppm): 6.52 (s, 4H, H_o), 6.70 (d, *J* = 3.6 Hz, 4H, H_l), 6.88 (t, *J* = 3.6 Hz, 4H, H_m), 7.10 (d, *J* = 3.6 Hz, 4H, H_n), 7.17 (s, 8H, $H_{j,k}$), 7.94 (d, *J* = 8.2 Hz, 1H, H_a), 7.99 (d, *J* = 9.2 Hz, 1H, H_d), 8.04 (t, *J* = 7.6 Hz, 1H, H_f), 8.08 (d, *J* = 8.9 Hz, 1H, H_i), 8.10 (d, *J* = 8.9 Hz, 1H, H_h), 8.16 (d, *J* = 9.2 Hz, 1H, H_c), 8.20 (d, *J* = 7.6 Hz, 1H, H_g) , 8.22 (d, *J* = 7.6 Hz, 1H, H_e) 8.24 (d, *J* = 8.2 Hz, 1H, H_b).

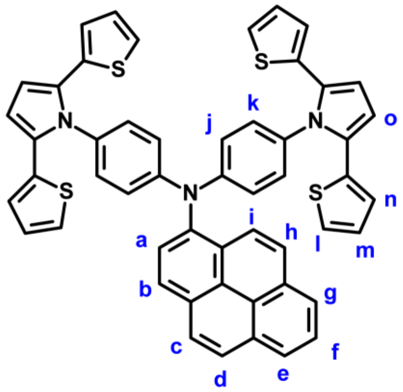

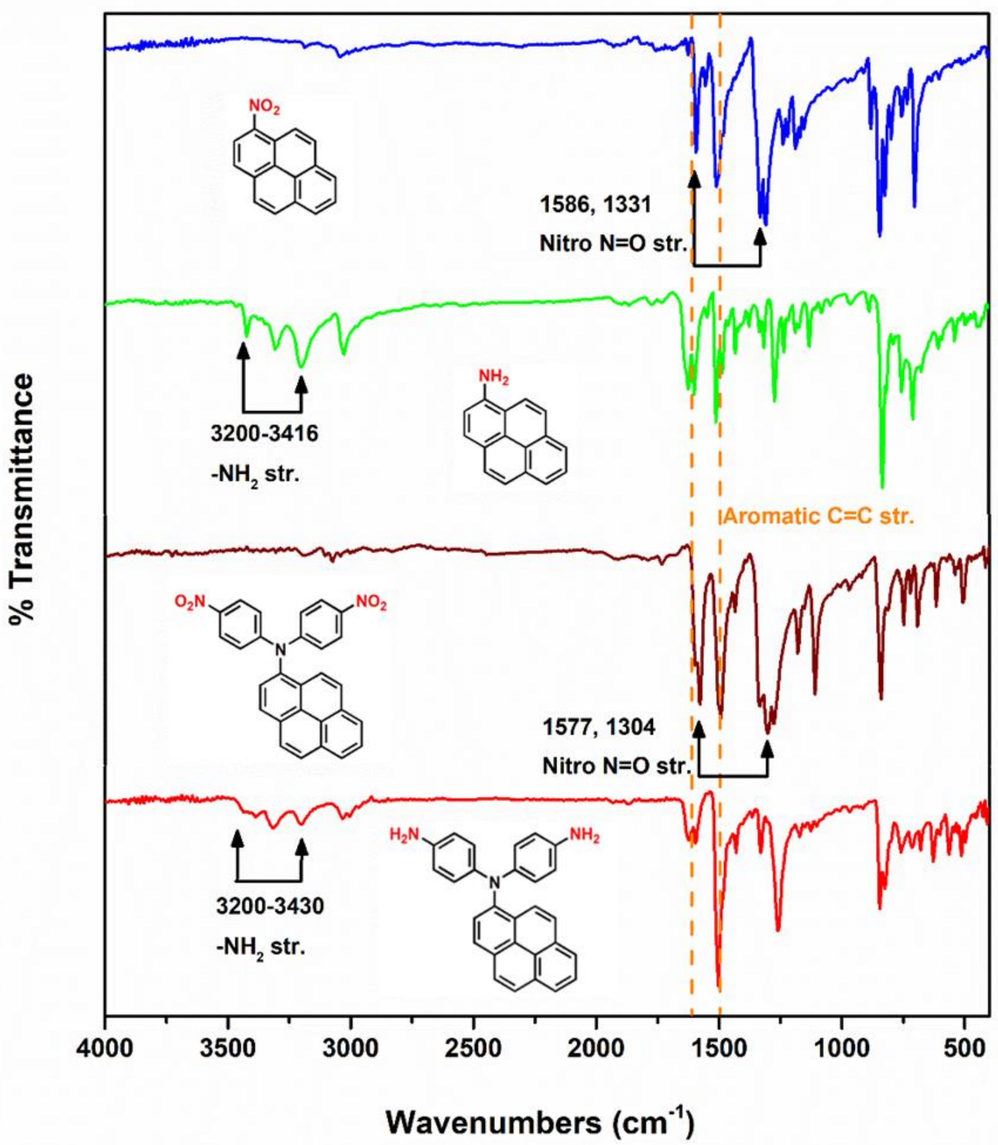

Figure S1. IR spectra of the 1-nitropyrene, 1-aminopyrene, *N*,*N*-di(4-nitrophenyl)-1-aminopyrene, and *N*,*N*-di(4-aminophenyl)-1-aminopyrene.

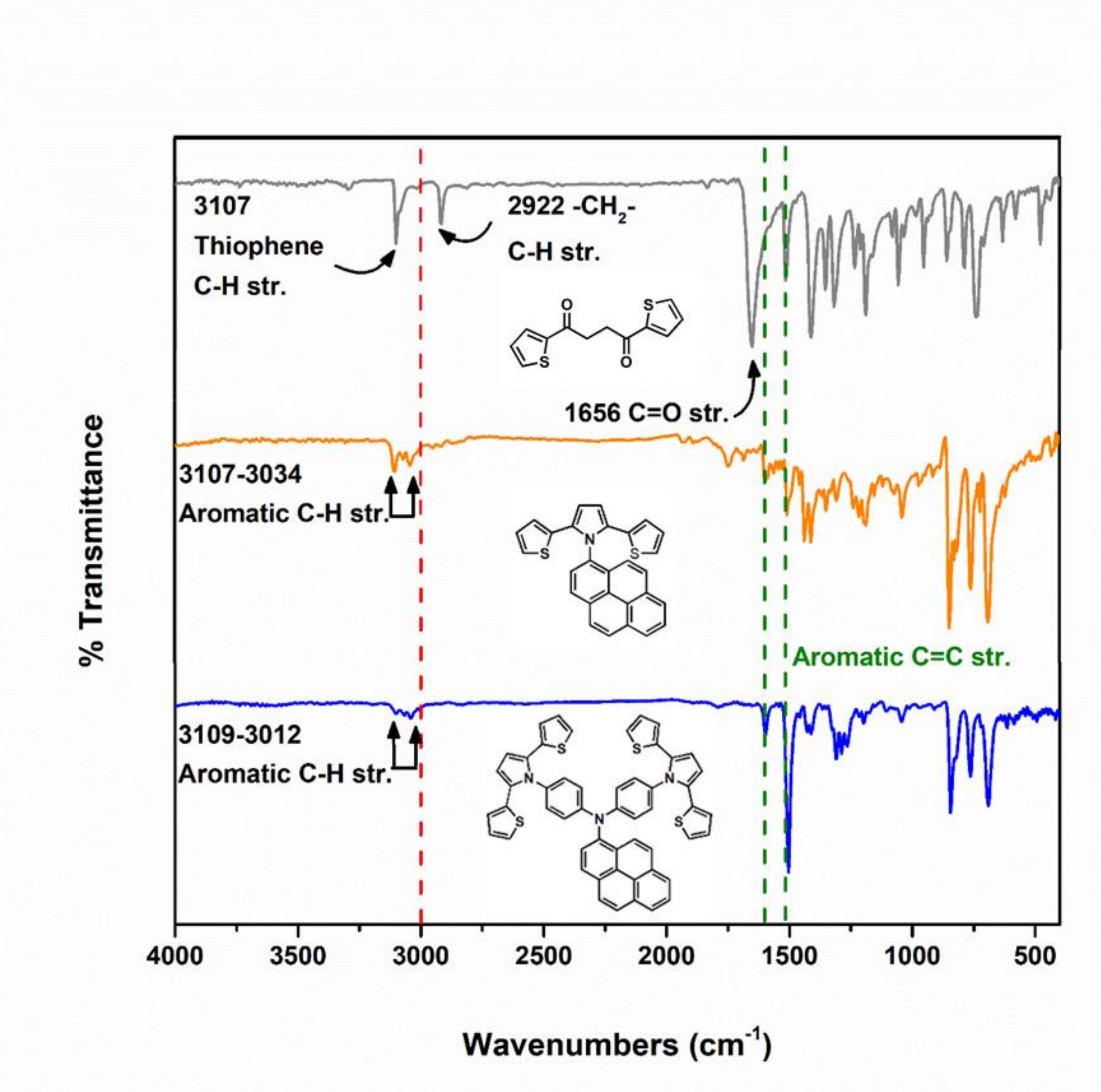

Figure S1. IR spectra of the 1,4-di(thiophen-2-yl)butane-1,4-dione, Py-SNS, and DPPA-2SNS.

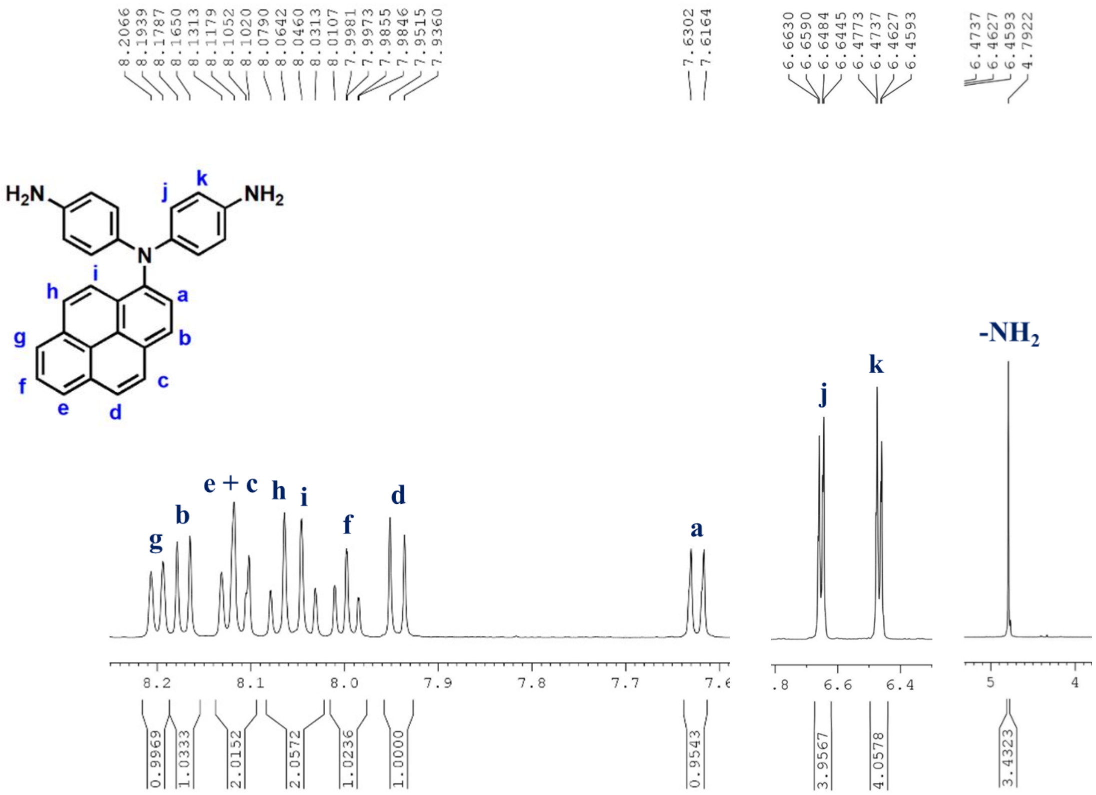

Figure S3. ^{1}H NMR spectrum of *N,N*-di(4-aminophenyl)-1-aminopyrene in DMSO-d_6.

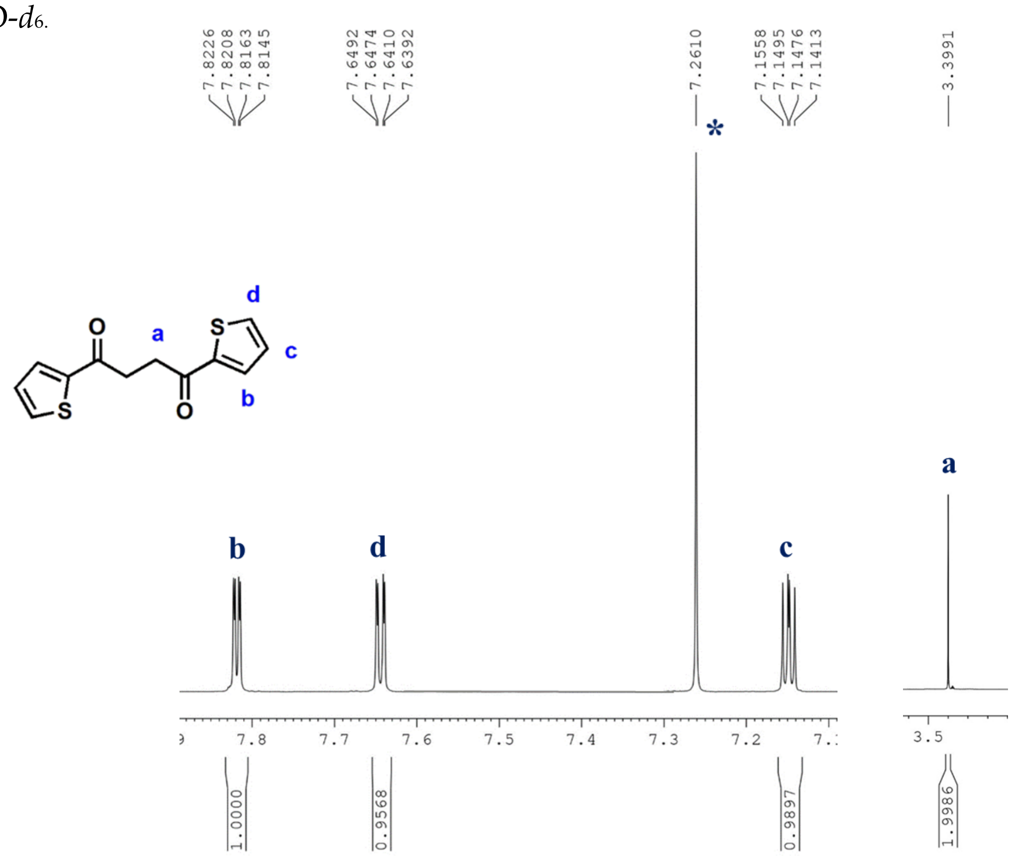

Figure S4. ^{1}H NMR spectrum of 1,4-di(thiophen-2-yl)butane-1,4-dione in $CDCl_3$ (* solvent peak).

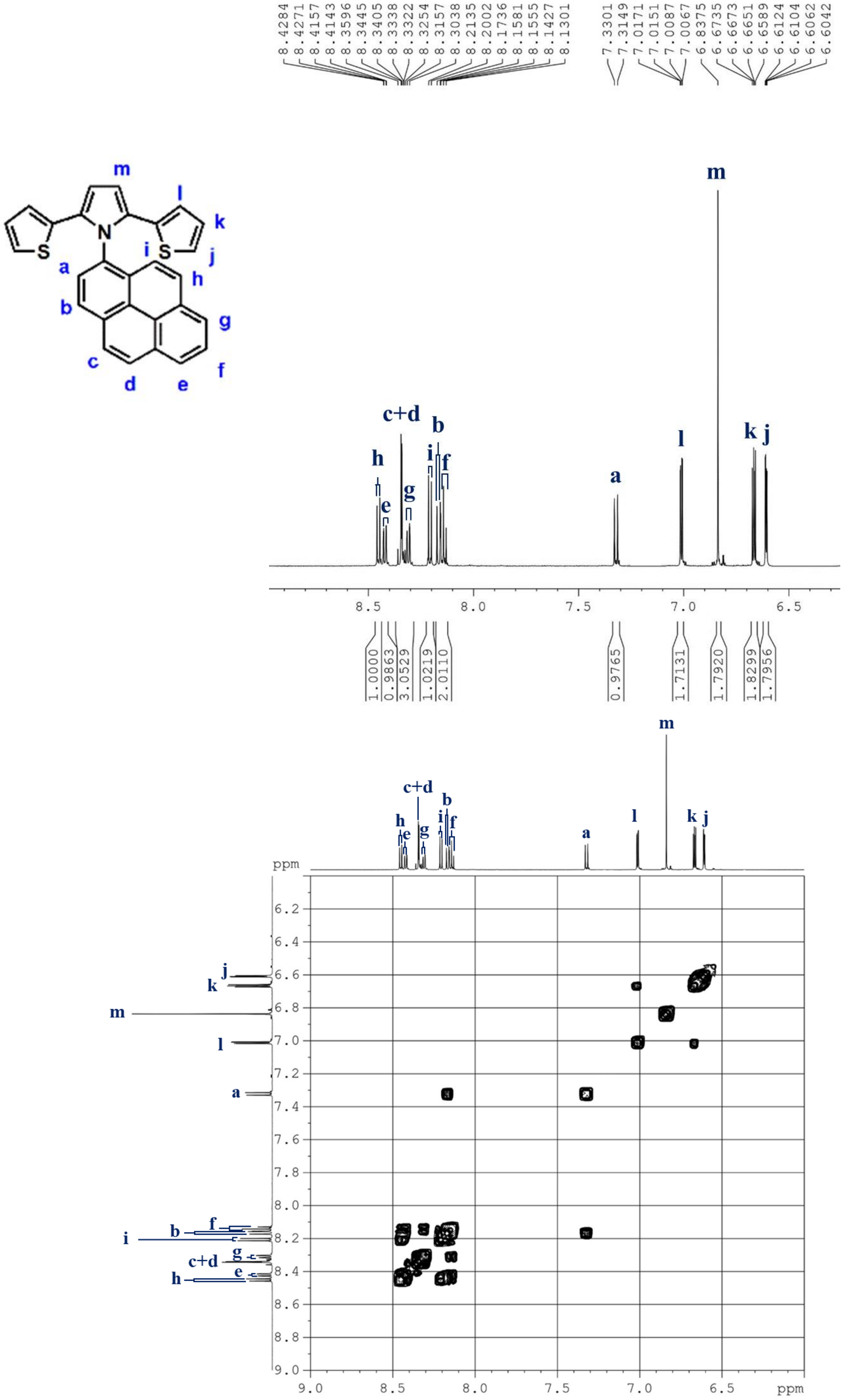

Figure S5. ^{1}H NMR and H-H COSY spectra of Py-SNS in DMSO-d_6.

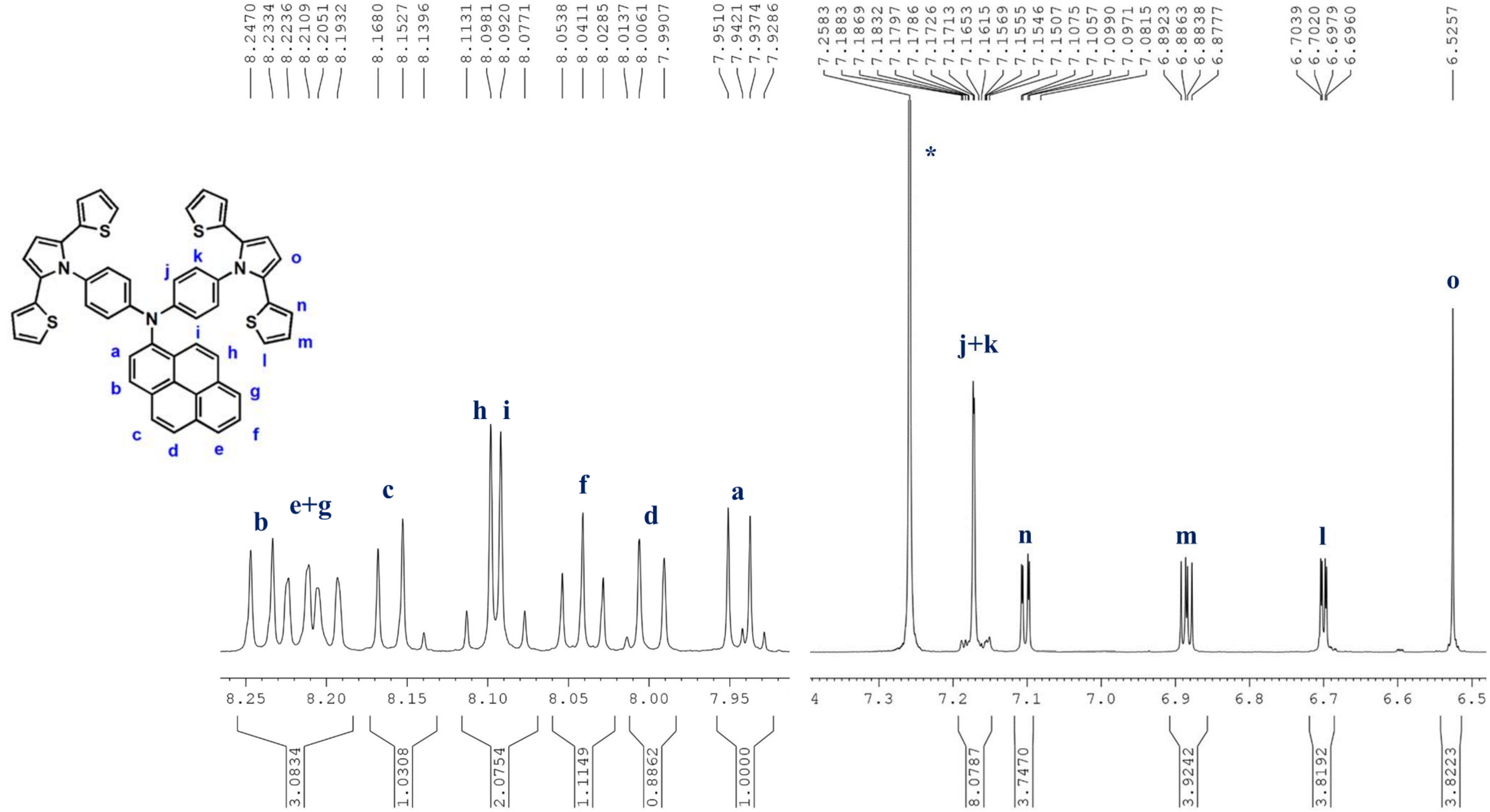

Figure S6. ^{1}H NMR spectrum of DPPA-2SNS in $CDCl_3$ (* solvent peak).

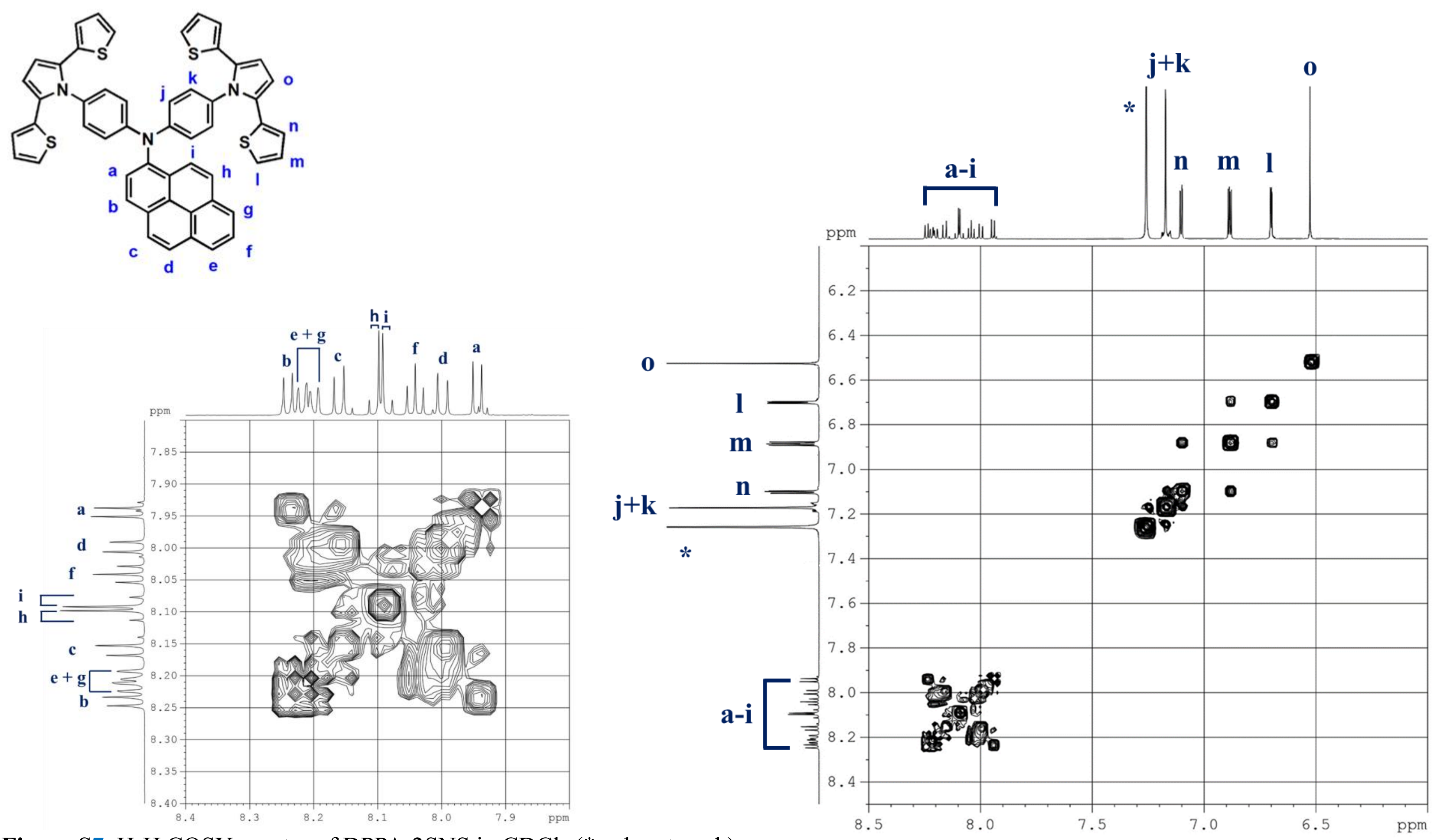

Figure S7. H-H COSY spectra of DPPA-2SNS in $CDCl_3$ (* solvent peak).

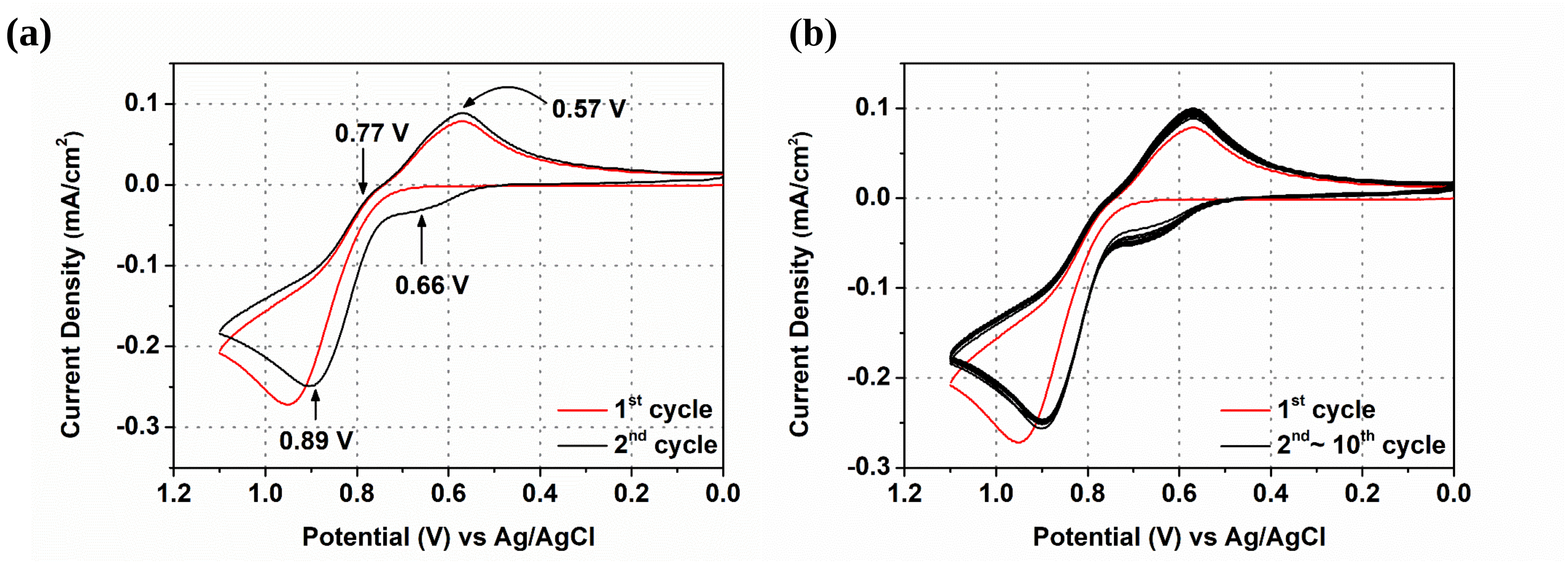

Figure S8. (a) The first CV scan, (b) the first two second CV scan, and (c) ten repetitive CV scans of 1 mM of Py-SNS in 0.1 M Bu_4NClO_4/CH_2Cl_2 in the potential range of 0.00–1.10 V at a scan rate of 50 mV/s.

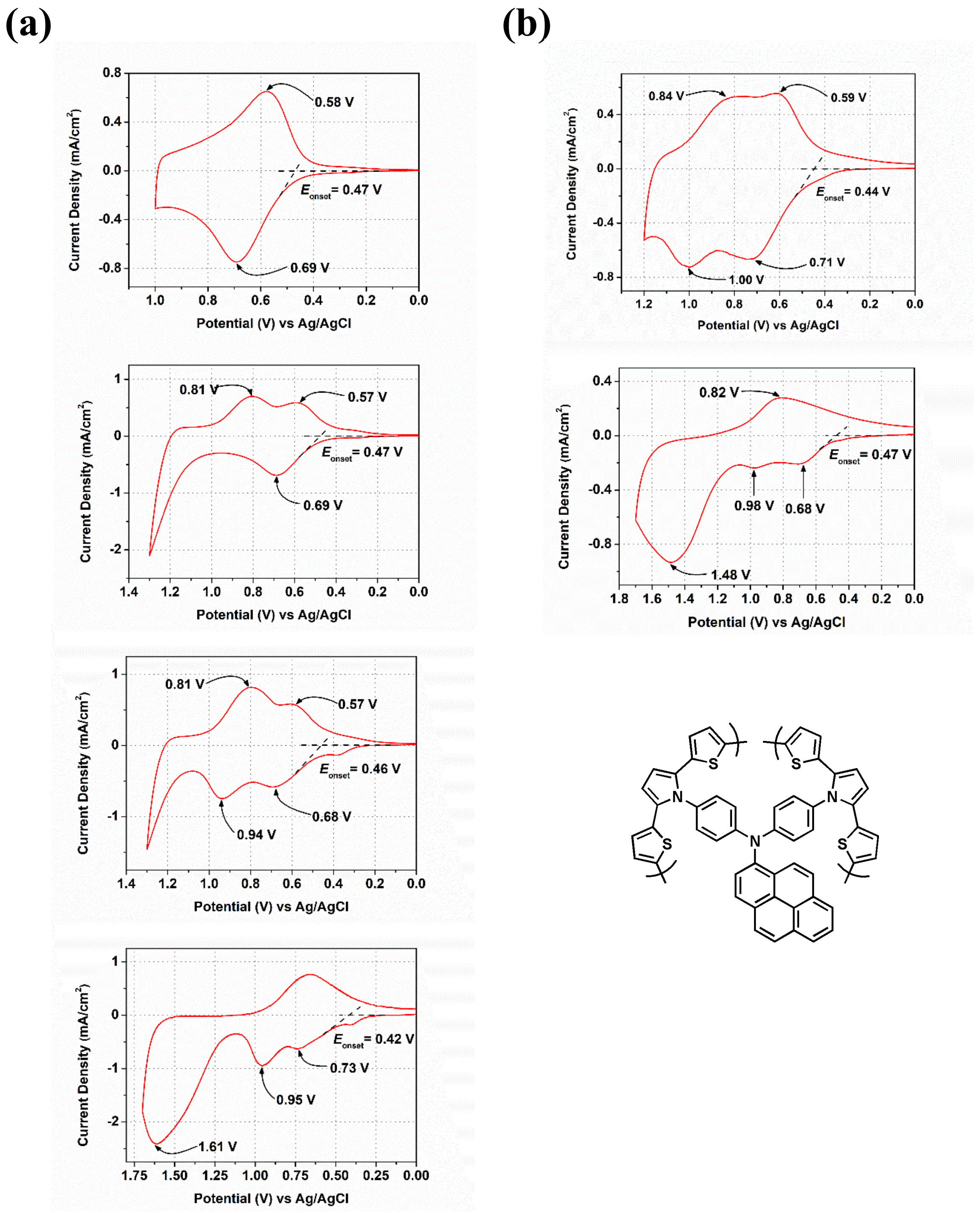

Figure S9. Cyclic voltammorgrams of the electrodeposited films of DPPA-2SNS on the ITO-coated glass slide in 0.1 M Bu_4NClO_4/MeCN at a scan rate of 50 mV/s. (a) Film A prepared by repeated CV scanning between 0 and 1.1 V and (b) film B prepared by repeated CV scanning between 0 and 1.3 V for ten cycles.

(a)

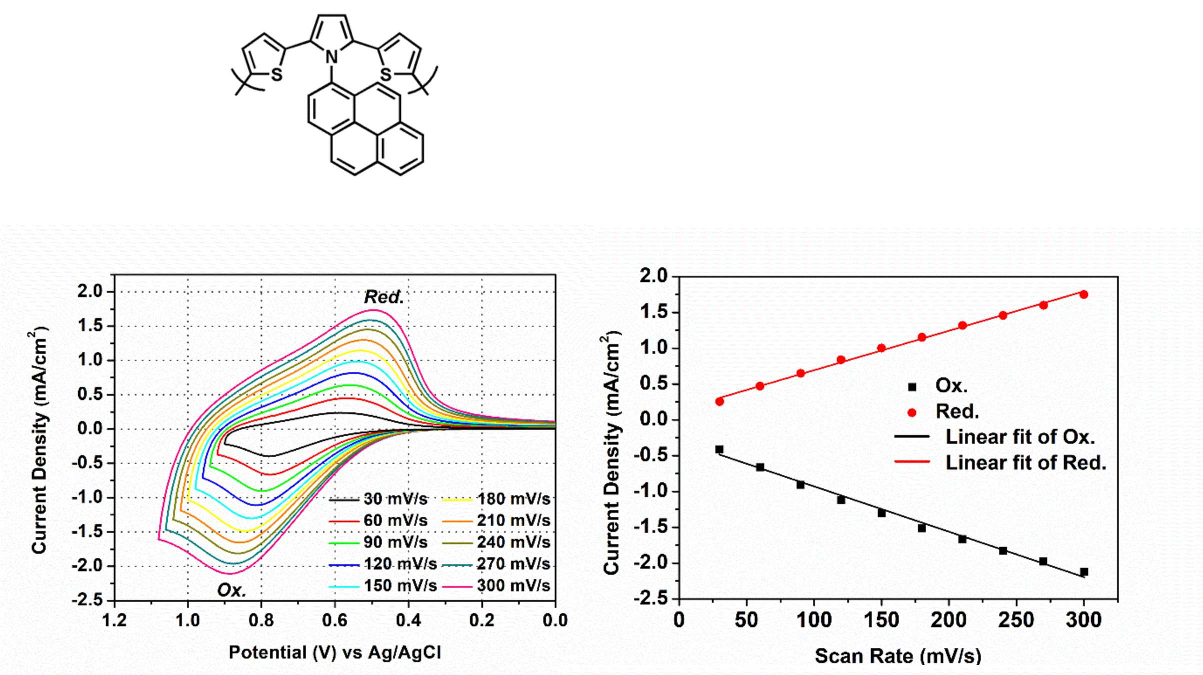

(b)

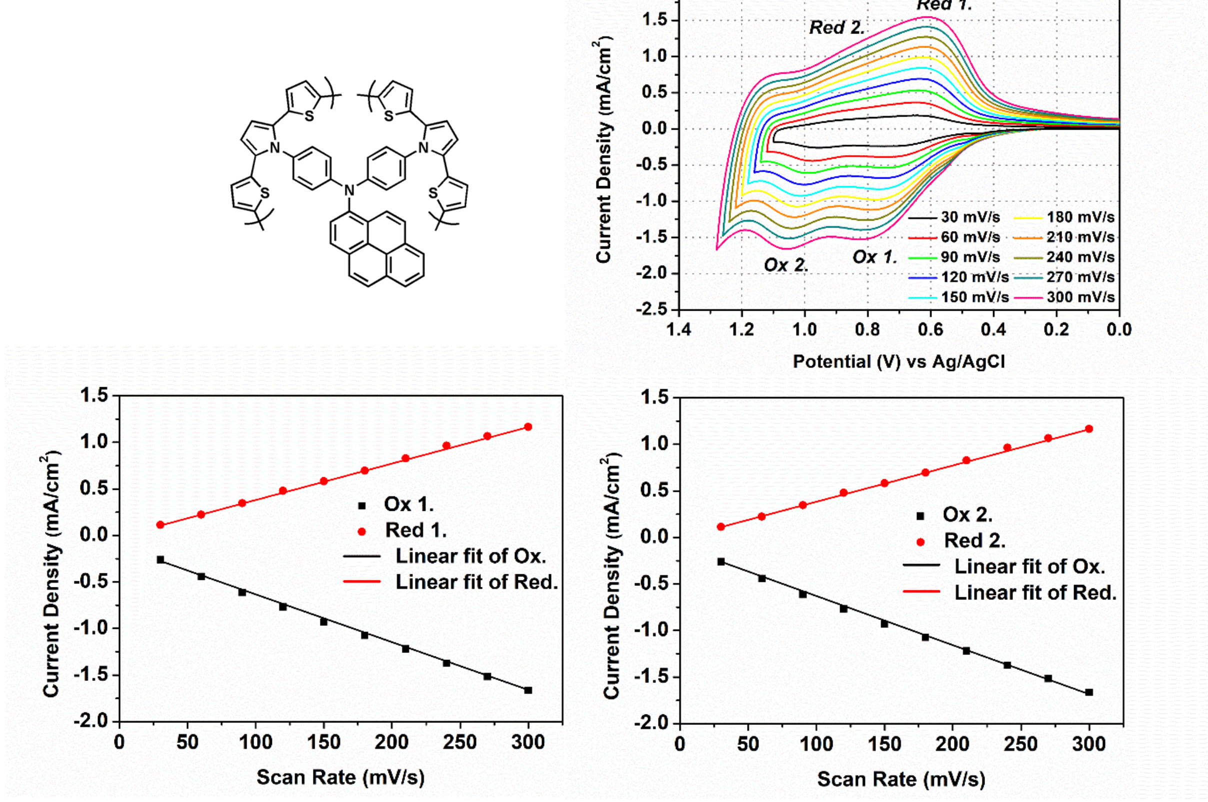

Figure S10. Scan rate dependence of polymer films (a) **P1** and (b) **P2** on the ITO-glass substrate in MeCN containing 0.1 M Bu_4NClO_4 at different scan rates from 30 to 300 mV/s.

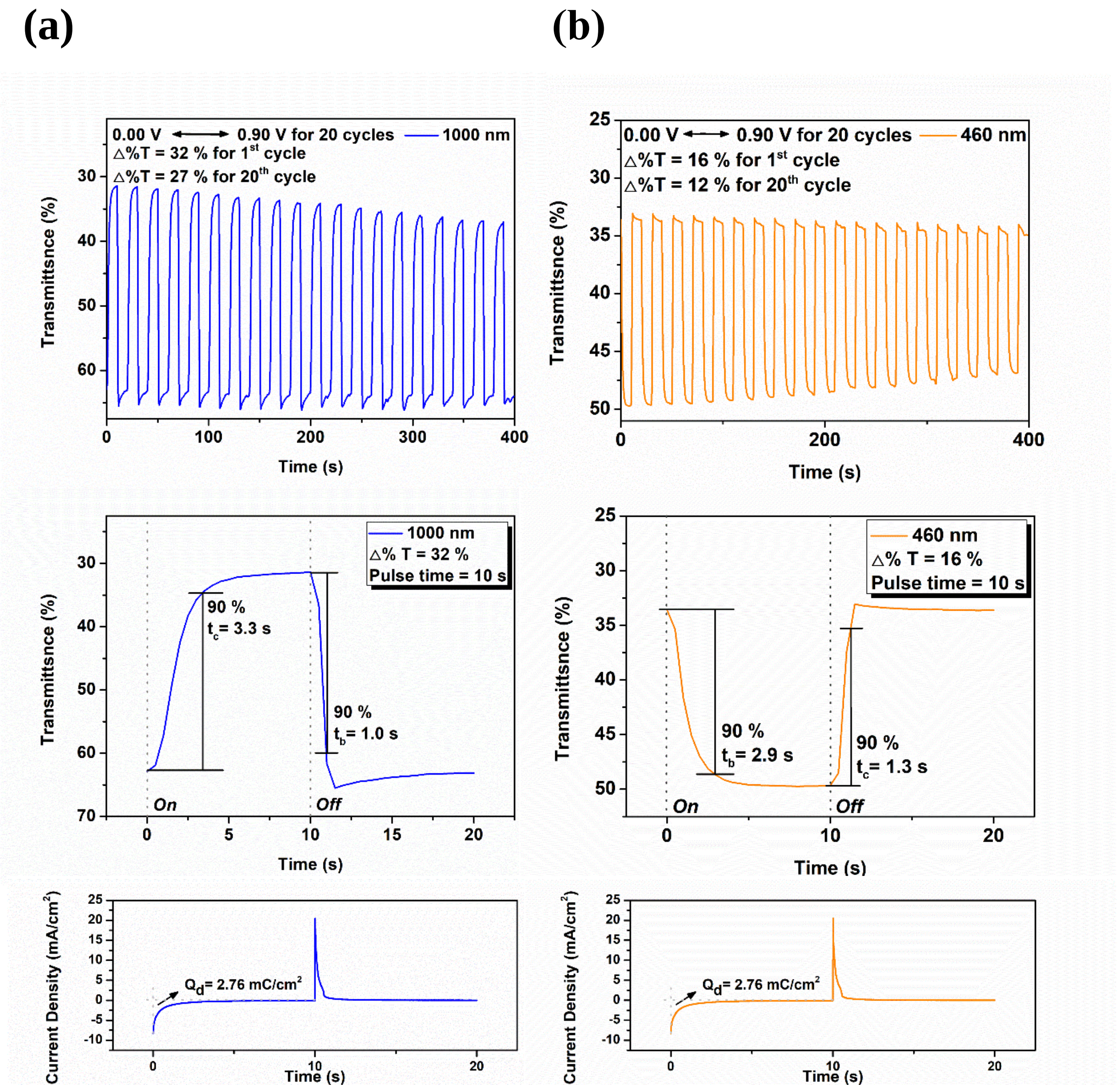

Figure S11. Potential step absorptiometry of the **P1** film (from Py-SNS) on the ITO-glass slide (in MeCN with 0.1 M Bu_4NClO_4 as a supporting electrolyte) by applying a potential step: (a) 0.00 V ⇆ 0.90 V (20 cycles) with a pulse width of 10 s at λ_{max} = 1000 nm and (b) optical switching at potential 0.00 V ⇆ 0.90 V (20 cycles) with a pulse width of 10 s at λ_{max}= 460 nm. The optical contrast and response times were calculated for the first switching cycle.

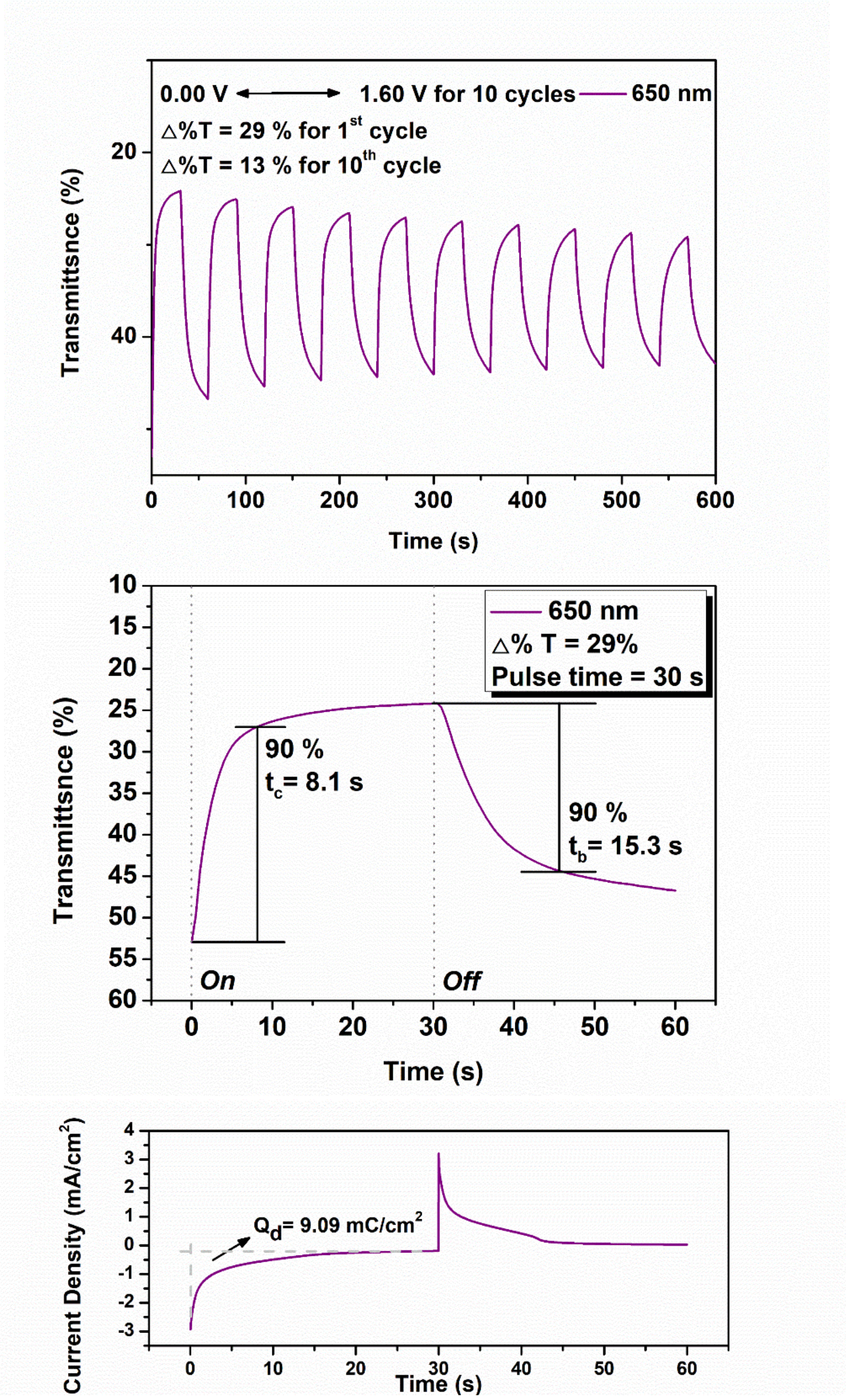

Figure S12. Potential step absorptiometry of the **P2** film (from DPPA-2SNS) on the ITO-glass slide (in MeCN with 0.1 M Bu_4NClO_4 as a supporting electrolyte) by applying a potential step 0.00 V ⇆ 1.60 V (10 cycles) with a pulse width of 30 s at λ_{max}= 650 nm. The optical contrast and response times were calculated for the first switching cycle.

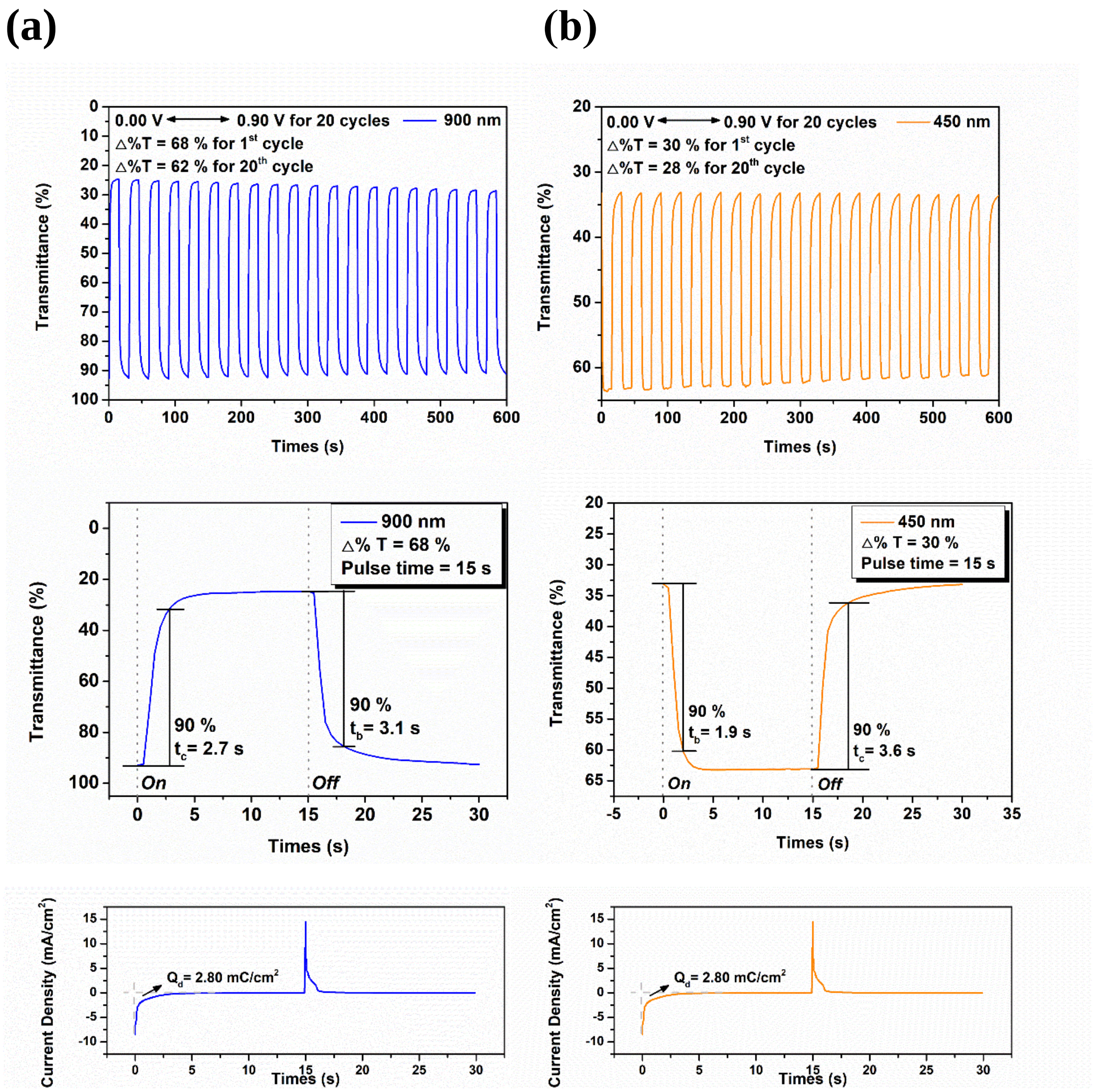

Figure S13. Potential step absorptiometry of the **P2** film (from DPPA-2SNS) on the ITO-glass slide (in MeCN with 0.1 M $LiClO_4$ as a supporting electrolyte) by applying a potential step: (a) 0.00 V ⇆ 0.90 V (20 cycles) with a pulse width of 15 s at λ_{max} = 900 nm and (b) 0.00 V ⇆ 0.90 V (20 cycles) with a pulse width of 15 s at λ_{max} = 450 nm. The optical contrast and response times were calculated for the first switching cycle.

Table S1. Optical properties of DPPA-2SNS in different solvents[a]

**Solvent**	ε^{b}	λ_{max}^{abs} **(nm)**[c]	λ_{max}^{PL} **(nm)**[d]	Φ_{PL} **(%)**[e]
Toluene	2.4	328	439	35
$CHCl_3$	4.8	327	447	5.1
THF	7.5	326	452	1.5
CH_2Cl_2	9.1	327	453	1.0
NMP	32.2	327	453	0.3
DMSO	47.0	328	453	0.2

[a] Measured at a concentration of ca. 1×10^{-5} M.

[b] Dielectric constant of the solvent.

[c] Wavelength at the absorption maximum.

[d] Excited at the absorption maximum for solution states.

[e] The fluorescence quantum yields determined by an integrating sphere, using 9,10-diphenylanthrance as a standard (Φ_{PL} = 90%, measured in dilute cyclohexane solution.)

Table S2. Electrochromic properties of the polymer films of **P2** with different electrolyte

Electrolyte	λ_{max}^{a} (nm)	Δ% T	Response time[b] t_c (s)	t_b (s)	ΔOD[c]	Q_d[d] (mC/cm²)	CE[e] (cm²/C)
Bu_4NClO_4/MeCN	900	58	4.7	4.6	0.46	2.05	224
	450	28	5.6	3.7	0.24	2.05	117
$LiClO_4$/MeCN	900	68	2.7	3.1	0.57	2.80	203
	450	30	3.6	1.9	0.28	2.80	100

[a] Wavelength of absorption maximum.

[b] Time for 90% of the full-transmittance change.

[c] Optical density change (ΔOD) = $\log[T_{bleached}/T_{colored}]$, where $T_{colored}$ and $T_{bleached}$ are the maximum transmittance in the oxidized and neutral states, respectively.

[d] Q_d is ejected charge, determined from the in situ experiments.

[e] Coloration efficiency (CE) =$\Delta OD/Q_d$.
